# Mouse ENU Mutagenesis to Understand Immunity to Infection: Methods, Selected Examples, and Perspectives

**DOI:** 10.3390/genes5040887

**Published:** 2014-09-29

**Authors:** Grégory Caignard, Megan M. Eva, Rebekah van Bruggen, Robert Eveleigh, Guillaume Bourque, Danielle Malo, Philippe Gros, Silvia M. Vidal

**Affiliations:** 1 Department of Human Genetics, McGill University, Montréal, QC H3G 0B1, Canada; E-Mail: gcaignard@gmail.com; 2 Complex Traits Group, McGill University, Montréal, QC H3G 0B1, Canada; 3 Department of Medicine, McGill University, Montréal, QC, H3G 0B1, Canada; E-Mails: megan.eva@mail.mcgill.ca (M.M.E.); danielle.malo@mcgill.ca (D.M.); 4 Department of Biochemistry, McGill University, Montréal, QC, H3G 0B1, Canada; E-Mails: rebekah.vanbruggen@mail.mcgill.ca (R.B.); philippe.gros@mcgill.ca (P.G.); 5 McGill University and Genome Quebec Innovation Center, Montréal, QC, H3A 1A4, Canada; E-Mails: eveleigh.rjm@gmail.com, (R.E.); guil.bourque@mcgill.ca (G.B.); 6 McGill Life Sciences Complex, Bellini Building, 3649 Sir William Osler Promenade, Room 367, Montreal, QC, H3G 0B1, Canada

**Keywords:** infectious diseases, ENU, immunity, mouse genetic models

## Abstract

Infectious diseases are responsible for over 25% of deaths globally, but many more individuals are exposed to deadly pathogens. The outcome of infection results from a set of diverse factors including pathogen virulence factors, the environment, and the genetic make-up of the host. The completion of the human reference genome sequence in 2004 along with technological advances have tremendously accelerated and renovated the tools to study the genetic etiology of infectious diseases in humans and its best characterized mammalian model, the mouse. Advancements in mouse genomic resources have accelerated genome-wide functional approaches, such as gene-driven and phenotype-driven mutagenesis, bringing to the fore the use of mouse models that reproduce accurately many aspects of the pathogenesis of human infectious diseases. Treatment with the mutagen *N*-ethyl-*N*-nitrosourea (ENU) has become the most popular phenotype-driven approach. Our team and others have employed mouse ENU mutagenesis to identify host genes that directly impact susceptibility to pathogens of global significance. In this review, we first describe the strategies and tools used in mouse genetics to understand immunity to infection with special emphasis on chemical mutagenesis of the mouse germ-line together with current strategies to efficiently identify functional mutations using next generation sequencing. Then, we highlight illustrative examples of genes, proteins, and cellular signatures that have been revealed by ENU screens and have been shown to be involved in susceptibility or resistance to infectious diseases caused by parasites, bacteria, and viruses.

## 1. Introduction

The Neolithic Era, which began around 10,000 years B.C., constituted a turning point in human civilization. Its importance stems not only from the establishment of the first human settlements, but also from the development of farming activities involving the domestication of wild plants and animals. These changes in societal organization brought humans into close contact with animals and soil, exposing them to potential new pathogens, and with each other, allowing the spread of any new infection. It therefore comes as no surprise that the Neolithic Era saw the emergence of several human infectious diseases [1]. Indeed, given this close proximity, trans-species infections became more likely and ultimately resulted in the appearance of diseases such as measles and smallpox [2]. As a result, from the Neolithic Era until the Industrial Revolution, human life expectancy did not exceed 25 years of age [3]. Fortunately, life expectancy has been steadily increasing over the last 150 years for two main reasons. First, public hygiene measures implemented in the mid-19th century reduced the transmission of infection. Additionally, the advent of vaccination and antimicrobial drugs in the late 19th and early 20th century meant that many deadly infections were now curable or preventable. On a larger scale, diseases such as polio and measles were drastically reduced, while the dreaded smallpox was completely eradicated.

**Figure 1:**
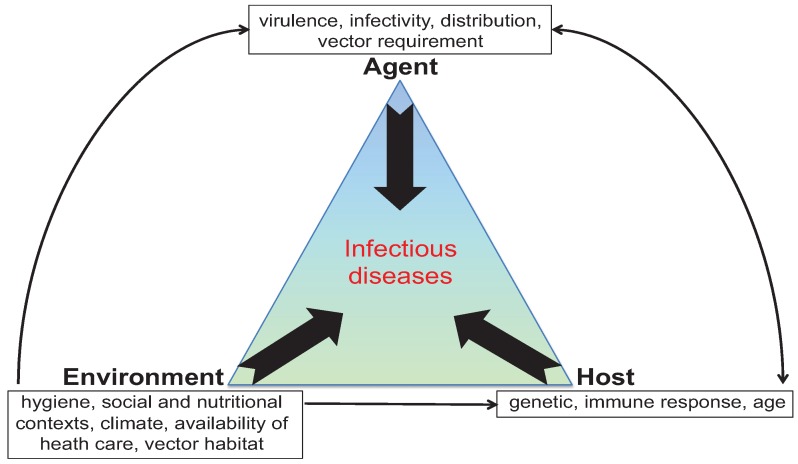
Factors involved in susceptibility to infectious diseases.

Nevertheless, infectious diseases remain directly responsible for close to 25% of all deaths globally and constitute a perpetual burden for humankind [4]. Numerous circumstances favor the emergence or reemergence of pathogens, or their spread to new ecological niches; these include pathogen virulence factors, as well as changing environmental conditions and host factors (e.g., aging populations, a heavier chronic disease burden, and therapeutic suppression of host defenses) (Figure 1). Of course, the eradication of most infectious diseases is highly unlikely. Instead, we are often involved in an unremitting struggle to control infection, for which a constant influx of novel countermeasure strategies is needed.

The development of these novel countermeasure strategies largely relies on a better understanding of the molecular mechanisms of disease pathogenesis. This requires not only basic research on the pathogen side but also on its interaction with the host. A possible approach is to exploit the observed variability in the outcome of infection, since at any given time, even during epidemics, clinical disease only develops in a subset of exposed persons. A large body of evidence indicates that the human genome is a major determinant of the variability in the onset, progression, and severity of infectious diseases [5,6,7,8]. In light of this evidence, research efforts aiming to better understand the pathogenesis of infectious diseases have shifted their focus from the pathogen to the host. Investigators are thus now attempting to identify host genes that are essential for successful pathogen infection, instead of focusing solely on pathogen genes. Candidate gene analysis studies have revealed a handful of single gene variants associated with increased susceptibility or resistance to specific infectious diseases (reviewed in [5]). Some remarkable examples identified in human populations include the malaria-protective effect of heterozygosity in the case of otherwise disease-causing hemoglobinopathies, such as sickle cell anemia and thalassemia [9], the protective effects of *CCR5* mutations against HIV [10], and resistance to norovirus infection conferred by loss-of-function alleles of the *FUT2* gene [11]. Further, the study of children with rare monogenic defects has revealed a considerable number of rare human genetic variations in innate immune pathways that underlie susceptibility to certain infectious diseases. For example, *IRAK* and *MYD88* deficiencies predispose to life-threatening infection by some bacterial species [12]. Another example is Mendelian Susceptibility to Mycobacterial Disease (MSMD), a primary immunodeficiency characterized by genetic defects in the IFNγ pathway, leading to susceptibility to *Mycobacterium bovis* (BCG) or other environmental mycobacteria species innocuous to the general population and to non-typhoidal, extra-intestinal salmonellosis (for review, see [5]). Thus, the fact that individuals exposed to life-threatening pathogens display differential susceptibility to infection and varying disease outcome not only reflects the genetic variability within the human population, but also the functional genetic diversity of the immune response itself.

The growing awareness of the importance of host genetic makeup in infectious disease outcome has motivated large-scale investigations of the human genome, made possible by recent technological advances. Namely, sequencing of the human genome [13], the International HapMap project [14], and microarray-based high-throughput genotyping technology have paved the way to Genome Wide Association Studies (GWAS) of major infectious diseases. In these GWAS, millions of single nucleotide polymorphisms (SNPs) can be tested for association with major infectious diseases, and this can be done simultaneously in thousands of individuals (for review, see [5]). Results emanating from these large datasets are certainly improving our understanding of infectious disease pathogenesis. However, full interpretation of the genes and pathways identified by GWAS studies is complicated by several factors including the modest effect size of most signals and the fact that even together these signals can explain only a fraction of the genetic predisposition to disease. Furthermore, the SNPs showing the strongest association are usually found near gene-coding regions rather than within obvious structural or regulatory regions making it difficult to pinpoint the gene directly involved in the disease phenotype. Such results are not entirely surprising given the inherent genetic heterogeneity of the human population, the variable exposure to the microbe during natural infection, the inherent variation in the microbe itself, and the difficulty associated with assembling the large cohorts required for GWAS. Yet, another key roadblock of GWAS studies is the lack of functional annotation for the majority of genes and encoded proteins, which is often limited to general ontology terms but lacks experimental validation for a possible role in an infectious disease phenotype.

## 2. Mice to the Rescue

An alternative and successful approach to identifying and characterizing the genetic component of the host response to infection in human studies has been the use of the mouse model. Owing to their striking physiological and genetic similarity with humans, mice have become a prime model for the study of human diseases. Numerous inbred strains exist that display natural resistance or susceptibility to a similar range of fungal, viral, parasitic, and bacterial pathogens, as well as the disease phenotypes associated with these infections [15,16,17,18]. These inbred strains represent homogeneous populations that serve to test different routes of inoculation, and various pathogen doses, all in a controlled environment, thus lessening many of the confounding effects encountered in human genetic studies. Due to its prominent role in biomedical research, the mouse was selected as the first non-human mammal to have its genome sequenced [19], revealing an astonishing genetic homology between the two species. The mouse and human genomes are approximately the same size, contain the same number of genes and show extensive conservation in gene order. Namely, 80% of human genes had 1:1 orthologous relationships with mouse genes, likely maintaining ancestral function in both species [20]. Mutations that cause diseases in humans often cause similar diseases in mice, including defects in the genes of the immune system [21]. Yet another advantage of the mouse is the string of unique technological advantages to manipulate the mouse genome. 

Using the mouse model, two major genetic approaches have been employed to dissect the genetic architecture of the host defense against pathogens. The first is the so-called reverse genetic or gene-driven approach. In this approach, the sequence or expression of a gene of interest is altered, the effects of which are then investigated. Genetic modification of the mouse genome can be undertaken in various ways: (1) transgenesis or the introduction of gene DNA sequences into oocytes; (2) targeted mutation using embryonic stem cells (ES) which are modified to create knock-out alleles, whereby the function of the gene is abolished and equivalent to a null allele, or knock-in alleles resulting from the introduction of putative mutations in a given gene. In addition, recently developed genomic resources have further facilitated the use of genetically modified mice by the scientific community. These include large libraries of knock-out and conditional knock-out mice produced by international consortia aiming to target every gene in the mouse genome [22] and their accompanying large-scale phenotyping initiatives [23]; (3) targeted mutation in zygotes using the Clustered regularly interspaced short palindromic repeat (CRISPR)/CRISPR associated (Cas9) system [24]. With this approach it is possible to efficiently produce mice with mutations in both copies of multiple genes in a matter of weeks [25]. The phenotypes of these genetically modified mice can then be thoroughly scrutinized to determine the function of a gene in the context of the whole organism. These tools are dramatically improving our understanding of the genetic etiology of infectious diseases in both mice and humans. However, in many instances, these reverse genetics experiments can prove to be inconclusive. This is the case, for example, when the inactivation of a gene results in embryonic lethality or, conversely, when the resulting phenotypes are only slightly different from the wild-type or even undistinguishable because of gene redundancy. The reverse genetics approach also requires a preliminary hypothesis for gene function. Yet, as of 2014, less than 50% of about 34,000 known mouse genes (coding or not) have some form of functional annotation based on experimental evidence [26,27,28], which shows how our understanding of gene function still lags behind our knowledge of gene sequence.

The second approach is known as forward genetics, sometimes called phenotype-driven. The forward genetics approach begins with an inherited phenotype, with the aim of identifying the genomic regions and variant(s) underlying it. This involves the production of segregating crosses of inbred mouse strains or panels of specialized strains that display varying responses to infection, followed by linkage or association analyses. This approach is unbiased and requires no prior knowledge of gene function, allowing the discovery of unsuspected mechanisms. Numerous laboratory mouse resources are readily accessible for use in these studies: homozygous inbred strains, panels of selectively bred strains, consomic strains [29], recombinant congenic strains [30,31,32] or recombinant inbred strains from the collaborative cross [33]. A growing number of wild-derived inbred strains [34] or outbred crosses [35] can also be obtained, increasing the pool of genomic variation available for these studies. Whole genome sequencing has been performed on 18 of the most commonly used inbred mouse strains; the results are now public [36,37], facilitating the identification of candidate genes underlying a given phenotypic variation. Moreover, forward genetics studies in mice have already been shown to work; some elegant examples have allowed the identification of a number of genes and proteins that are essential for the early detection of and response to many invading pathogens (for review see [38]). In some cases, the human orthologues of these mouse genes (e.g., *NRAMP1*, *TLR4*, *IRF8*) have also been associated with predisposition to infection in humans, providing evidence of evolutionary conservation of immune defense mechanisms. However, there are limitations to this forward genetics strategy. Namely, a given genetic effect may be complex, making it difficult for investigators to determine the contribution of individual genes, as this requires subsequent breeding of congenic mice over several generations followed by positional cloning. Identifying the precise nature of a genetic lesion in a given candidate gene can also be complicated for other reasons, such as the presence of multigenic families or unrelated genes within the candidate interval bearing various coding polymorphisms, or predictive regulatory mutations or splicing variants rendering it difficult to identify the causative variant. Many of these drawbacks, however, can be overcome by the use of mutagens that introduce random mutations in the germ line. As presented later, in these models the causative mutation can be more easily identified by comparison with the parental non-mutagenized strain. This functional genomic strategy has successfully advanced our understanding of the intricate cellular and molecular cascades involved in immunodeficiency, autoimmunity, or behavioral disorders, which have already been well documented by others (see [39,40,41,42]). In the remainder of this review, we present the advantages and how-to of experiments using chemical mutagenesis of the mouse germ-line to dissect the genetic architecture of immunity to infection in mice. We also detail the procedures required to identify causal mutations underlying altered phenotypes using next generation sequencing. Finally, we highlight some of the most important findings from *in vivo* screens in the area of infectious disease research and discuss perspectives for mouse ENU approaches.

## 3. Chemical Mutagenesis and Generation of Mice Carrying Homozygous ENU-Induced Mutations

To better understand the link between genotypes and phenotypes, and ultimately gene function, mouse geneticists have elaborated upon several methods capable of introducing random mutations in the mouse germ-line, with the aim of expanding the phenotypic diversity in inbred mice and thus providing a wider range of research objects. These methods include the use of whole mouse radiation [43], infection of pre-implantation embryos with retroviruses [44], and injection with chemicals, such as procarbazine, methyl ethane sulfonate (MES), and *N*-ethyl-*N*-nitrosourea (ENU) [45]. ENU mutagenesis, however, has become the most popular technique to induce germ-line mutations due to its advantageous attributes: potency, preferential activity in spermatogonial stem cells, and a propensity to introduce point mutations.

As early as 1979, W. L. Russell demonstrated that a single dose of ENU was significantly more active than X-ray or procarbazine treatment, the most commonly used mouse mutagens at the time [46]. Later, studies showed that the mutation frequency could be increased if the ENU dose was fractionated and injected on a weekly schedule instead of being administered in one large dose, as this allowed a higher total dose to be tolerated [47]. In these conditions, the activity of ENU was 12 times that of X-rays and 36 times that of procarbazine, as well as being over 200 times the rate of spontaneous mutation [48]. The rate of ENU mutation appears variable for each gene, ranging from 1.5 to 10^−3^ per locus, which is equivalent to obtaining a mutation in a gene of choice at a rate of one in every 200–700 gametes screened. Additionally, it was noted that compared to X-ray-generated deletions, ENU rarely induced mutations in closely linked loci, suggesting that mutations introduced by ENU are subtler. Finally, compared to procarbazine, which is more active in transient post-meiotic cells, ENU preferentially affects spermatogonial stem cells, which are multiplied and replenished during the mouse lifetime, allowing the genetic lesions to be recovered indefinitely.

ENU is an alkylating agent that acts by preferential transfer of its ethyl group to O and N radicals in genomic DNA within mammalian cells [49,50]. Binding of the ethyl to the nucleoradicals creates DNA adducts that provoke mispairing, resulting mainly in base-pair substitutions if not restored by enzymatic DNA repair mechanisms during replication [51,52]. Systematic analysis of the type and frequency of ENU mutations was recently done using whole-exome and whole-genome sequencing [53,54,55]. Genome-wide, ENU has an average point mutation rate of 1.5 per Mb of genomic DNA [55], with a bias for AT to GC transitions (45%) compared to AT to TA transversions (28%). The size of a given target gene and its AT density can therefore explain, at least in part, the variable sensitivity to the mutagenic effects of ENU. With a mouse genome size of about 2.7 Mb including 1.5% of protein coding sequence, one can expect about 1,900 new sequence variants per genome of which about 30 are coding.

With a few exceptions (microRNA and cis-elements) [56,57], to date most ENU-induced phenotypes, whose corresponding genotype has been identified, result from nucleotide changes that alter the coding sequencing. A current survey of the Mutagenix database (http://mutagenetix.utsouthwestern.edu/home.cfm) which contains the largest collection of ENU-induced phenotypic mutations (N = 185) [58], revealed that 61% were missense mutations, 19% nonsense alleles, 18% splicing defects, and 2% were frame-shift mutations. Therefore, while targeted mutations producing null alleles are necessary for genetic dissection of phenotypic traits, ENU-induced point mutations can also be used in parallel, revealing the multiple functions of a gene by altering individual protein domains and splicing products. Further, point mutations can produce various types of allelic series: (1) hypermorphic or hypomorphic alleles (increased or reduced activity of the gene product, respectively); (2) antimorphic alleles (the gene product is antagonistic to the wild-type allele); or (3) neomorphic alleles (new molecular function) [59] which can display a broad range of possible phenotypes. 

The phenotypes that arise following ENU mutagenesis segregate with different inheritance patterns. Autosomal recessive (68%) is the most commonly observed, followed by dominant or co-dominant segregation (23%); X-linked recessive (4%) or X-linked dominant (1%) are rare, though 4% remain uncharacterized [58]. Once male mice have been treated with ENU, they are crossed to female mice. The resulting large cohorts of offspring are then tested to identify the phenotypically distinct mice most likely to bear a large-effect mutation; this is usually done with dominant or recessive screens. The above data illustrates how recessive screens, which require a three-generation breeding scheme (see below), constitute a more efficient and inclusive design than dominant screens, although the latter are logistically simpler and quicker to conduct since only the first generation offspring are analyzed. Using different breeding schemes, these recessive screens have successfully advanced our understanding of the intricate cellular and molecular cascades involved in immunodeficiency and autoimmunity, as well as in neurological or behavioral disorders, as already reviewed by others (see [39,40,41,42]).

**Figure 2:**
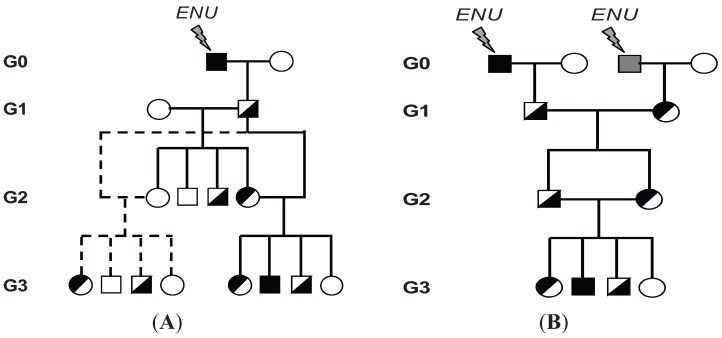
Breeding strategies used in our program to produce mice carrying homozygous *N*-ethyl-*N*-nitrosourea (ENU) mutations. (**A**) Treatment with ENU introduces mutations (indicated by a black or gray square) in the germ-line of males of generation 0 (G0). The mutagenized G0 male is out-crossed to a wild-type female to produce first generation (G1) animals. First generation G1 mice are carriers of ENU-induced mutations (indicated by half-filled black squares). G1 males are mated to wild-type females, to produce second generation (G2) animals, which carry about half of the mutation load present in the parental G1. Two G2 daughters are backcrossed to their G1 father to yield third-generation (G3) mice, where the original mutations have been brought to homozygosity (filled black squares). About 25% G3 progeny are expected to present a deviant phenotype in pedigrees that bear a given relevant recessive mutations; (**B**) In this strategy, the G1 progeny of two independent G0 males are intercrossed to produce G2 animals, which in turn are intercrossed to produce G3 mice.

Methods for mutagenizing male mice and breeding protocols to recover homozygous mutations have been described previously [60,61,62]. In our laboratory, we use a recessive screen involving genetically related mouse strains to generate the collections of mutant mice (Figure 2). By using genetically related inbred strains, the number of animals used and the timeline of the experiment can be reduced, as the mice that are screened also serve for mapping of the ENU-induced phenotypes. Moreover, using closely related strains alleviates any possible second-site modifier gene effects that could be present in the mapping strain. Briefly, we use well-validated protocols to induce single nucleotide mutations in 129S1/SvImJ (129S1) and C57BL/6 (B6) mice. This is done using a single intraperitoneal (i.p.) injection of 150 mg/kg of ENU (129S1) or three weekly i.p. injections of 90 mg/kg (B6) [63]. Following treatment, spermatogenesis ceases transiently and fertility is then regained after 11–13 weeks. In a general breeding strategy (Figure 2A), generation 0 (G0) males are then out-crossed with wild-type female mice to produce G1 offspring. These G1 hybrids carry one full set of mutagenized chromosomes and one full set of wild-type chromosomes. Individual G1 males are bred as founders of separate pedigrees, with the aim of bringing B6 or 129S1 sequence variants to homozygosity. To achieve it, G1 males are first crossed with genetically related wild-type females (129X1/SvJ (129X1) females for 129S1 males and C57BL/10 (B10) females for B6 males) to distinguish mutation-bearing chromosomes while preventing the introduction of additional genetic modifiers. The mutations present in the G1 founders are thus propagated in the G2 progeny. Since each G2 offspring should inherit only 50% of sequence variants present in the G1 males, two G2 daughters are backcrossed to their G1 father. This produces G3 progeny, where 12.5% of the G1 sequence variants should come to homozygosity in any given G3 offspring. On average, each G3 offspring is thus expected to be homozygous for about four to five loss-of-function sequence variants of the 30 present in the G1. Therefore, if there is a recessive Mendelian immune variant segregating within a pedigree, researchers can expect to identify 25% of individuals with the same trait or a cluster of two to four deviants by initially screening about 16 G3 offspring in that pedigree. The clustering of heritable variants within a pedigree filters out unavoidable false positives, which occur at a low rate (~5%) in screens for host susceptibility to infection; typically only one individual constitutes a false positive in a given pedigree. Variations of this breeding strategy have been used (Figure 2B) and will be described in the corresponding sections.

This pedigree structure allows early mapping of heritable variants. At this point, breeding and screening of additional G3 siblings confirm the inheritance of Mendelian recessive infectious traits in one quarter of the offspring. If eight to ten G3 animals displaying a new recessive immune trait are obtained out of 40–50 G3 mice in the pedigree, a genome-wide scan can be performed to establish linkage of the variant to a large initial segment. Before the advent of next generation sequencing (NGS), a time consuming and labor intensive positional cloning approach had to be undertaken to identify candidate genes bearing new genetic variants. The use of NGS techniques has dramatically increased the pace of mutation identification.

## 4. Gene Identification

The materials and methods underlying phenotype-driven or forward genetics approaches have become considerably more powerful over the years. Traditionally, these approaches required laborious genetic and fine mapping procedures in order to refine regions of interest to large megabase (Mb) chromosomal loci for subsequent PCR amplification and direct sequencing. Nonetheless, they were the methods of choice for the discovery of novel genes and/or novel gene functions in both humans and mice. The introduction of NGS has revolutionized forward genetics approaches, as it allowed the elaboration of robust methods of systematic mutation discovery, thus further closing the gap between phenotype and genotype. However, the sequencing and analysis of whole mammalian genomes remain a substantial bottleneck for many laboratories, both financially and computationally. Instead, inexpensive alternatives have been favored in order to sequence mouse mutations, namely targeting approaches using minimal mapping data. Moreover, targeted sequencing of coding regions of the genomes, or exomes, are particularly relevant for large mutational collections and have become the standard in cases where high-throughput gene mutation discovery methods are needed [64,65,66]. We describe below some of the standard techniques for sequencing and analysis of *de novo* mutations generated within ENU mouse models in a rapid and unbiased fashion.

Currently, the most widely used commercial mouse exome capture panels (Agilent and NimbleGen) target approximately 37 Mb of the sequences contained within the consensus coding sequence (CCDS) database of the genome, as well as other genomic features (e.g., microRNAs) [53,67] (see Table 1). The protocols contained in each of these kits are very similar. First, labeled DNA (or RNA) baits ranging from 55 to 120 bases are hybridized to fragmented genomic DNA. The baits are pulled down using magnetic beads, and the “captured” genomic fragments are then sequenced using NGS instruments such as SOLiD, Illumina or Roche 454. 

**Table 1:** Comparison between two standard whole exome mouse capture kits.

	Agilent Sureselect Mouse All Exon	Nimblegen SeqCap Ez
Probe size	120 bases	55–105 bases
Target Region size	49.6 Mb	54.3 Mb
Probe Type	RNA	DNA
Number of Targeted Exons	221,784	203,225

Mutation identification and ultimately gene discovery in the context of ENU-designed projects require significant computational analyses, where sequenced DNA fragments are mapped to a mouse reference sequence (C57BL/6J) [68] or to that of a specific mouse strain when available [36], followed by post alignment and variant calling procedures. For a given mouse sample, these procedures typically produce a large amount of single nucleotide variants (SNVs) and insertion/deletions (INDELs), which, depending on the sample’s genetic background and coverage, can range from a few thousand to hundreds of thousands in more divergent strains. Further steps are required to filter the strain specific variants if the reference sequence of the mouse background is not used. This can be accomplished, for example, by adding more controls. 

**Figure 3:**
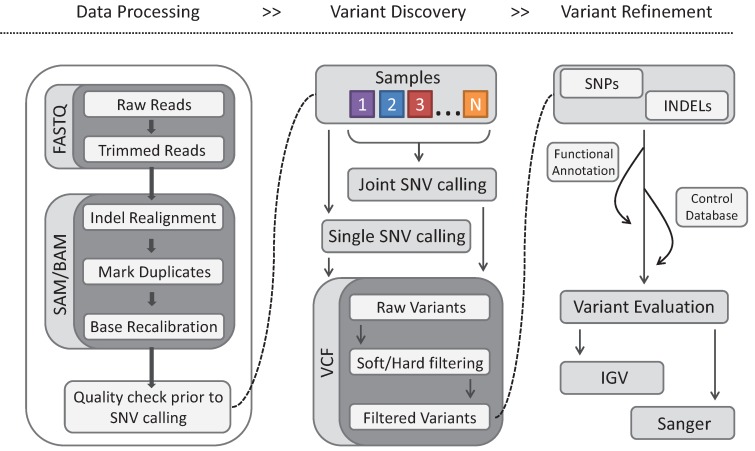
A typical workflow to identify causative mutations in genomic data. The procedures are separated into three general processes: (1) data processing, where raw sequencing data (fastq format) is aligned (sam/bam file format) to a known genome reference followed by alignment improvement steps (*i.e*., indel realignment, mark duplicates and base recalibration); (2) a variant discovery step in which single nucleotide variants (SNVs) are called from aligned data followed by subsequent filtering (using variant quality thresholds; hard filtering, or Genome Analysis Toolkit (GATK) variant recalibration; and soft filtering); (3) and a variant refinement step to reduce the number of candidate mutations to a manageable number for further validation using Integrative Genomics Viewer (IGV) and/or Sanger sequencing [71].

Numerous workflows (e.g., Genome Analysis Toolkit (GATK) best practices [69] and McGill University and Genome Quebec Innovation Centre (MUQGIC) [70]) have been designed for mutation discovery. Although each design may vary with regards to the steps and computational programs utilized, the underlying principle of these workflows remains the same. Each one divides the processing and analysis of sequencing data into three key steps: (1) data processing for quality control and filtering of sequenced reads; (2) variant discovery through alignment of filtered reads to known reference genomes; and (3) variant refinement leading to variant calling to identify mutations of interest. A flow diagram similar to GATK best practices [71] but with subdivided steps in file format is shown (Figure 3).

The sequenced reads (in fastq file format) are usually derived from the instrument specific base-calling algorithm (or subsequent steps therein) and contain an identifier for each raw DNA fragment, as well as a phred quality score for each base in the fragment. The raw reads are aligned to a reference genome following a quality control step or “trimmed” to obtain a high quality set of reads for sequence alignment file (sam/bam) generation. The trimming step removes adaptor sequences from the raw reads and optionally removes bases at the 3' end using a specified phred quality threshold, and/or performs a size selection filtering step (e.g., trimmomatic [72]; Figure 3). The trimmed reads are aligned by using either a “hashing” or an effective data compression algorithm called the “Burrows-Wheeler transform” (BWT). Fast, memory-efficient BWT-based aligners, such as BWA [73], are often used in NGS studies. However, these aligners tend to be less sensitive than recent hash-based aligners, such as Novoalign [74], which conversely tend to require more computational resources [75].

Numerous software packages such as GATK [69], samtools [76], and Picard [77] have been developed to attempt to correct for biases incorporated at the sequencing and alignment phases, thus improving variant detection (Figure 3). During library construction and sequencing, duplicated DNA fragments produced by polymerase chain reaction (PCR) amplification and optical duplicates can occur. Software package such as Picard markDup and Samtools rmdup remove or flag potential PCR duplicates if both mates (in the case of paired-end reads) contain the same 5' alignment positions. 

At the alignment phase, due in part to the heuristics of the alignment algorithm and the alignment scoring procedure, refinement of mapped reads near indels (GATK indel realigner [69]) and quality scores (GATK base recalibration [69]) are typically required to help reduce false positive variants in downstream analysis. Utilizing these two post-alignment programs, GATK indel realigner transforms regions with misalignments generally introduced by indels into clean reads containing fewer mismatches, whereas base recalibration improves the quality score to better reflect the true base-calling error rates by correcting for variation in quality with respect to machine cycle, sequence context, and other attributes.

To identify the protein-encoding mutations induced by ENU, numerous variant-calling procedures can be employed to convert base calls and quality scores into a set of genotypes on a per sample basis. The most recent variant callers, such as GATK [69], Samtools [75], and FreeBayes [78], use sophisticated statistical models that can be extended to incorporate additional information regarding allele frequencies and/or linkage disequilibrium (LD) patterns. Furthermore, joint analysis of multiple individuals can further improve genotype calling for single samples by taking into account allele frequencies or genotype frequencies [79].

Variant detection programs convert the refined base-calls and quality scores resulting from the post-alignment process and generate variant data containing information regarding the genomic position, SNV quality, *etc.*, of each variant. Generally, thousands of SNVs are generated by the detection protocol. Further annotations and filtering procedures are thus required to identify the expected 50–100 ENU-induced mutations [80]. The use of functional annotation programs such as snpEff [81] and VEP [82], coupled with the exclusion of known variants (for example, on the basis of SNP data from the dbSNP database [83]) and of variants falling below acceptable quality metrics (QUAL, genotype quality (GQ), strand bias, *etc.*), can help to preferentially identify protein coding mutations. However, despite rigorous post-alignment refinement and variant exclusion criteria, recurrent false positive SNVs remain. By comparing a set of ENU samples to unrelated genome or exome sequencing data sets, as well as to mouse genomes data from the Sanger Institute [68] generated using the same analysis workflow, variants commonly shared between related strains or systematic false positives arising from mapping issues related to genome structure (e.g., repetitive or paralogous sequences) or errors (e.g., miss-annotated reference allele) can be flagged for removal. In numerous studies this procedure has proven successful in prioritizing candidate mutations and decreasing their numbers [54,80], and has helped reduce the time requirements and cost of visual inspection (e.g., Integrative Genomics Viewer (IGV) [84]), of Sanger sequencing [85], of validation, and ultimately of novel mutation/gene discovery.

ENU experiments have successfully identified candidate causative mutations residing in protein coding sequences, splice sites or UTRs. However, these causative mutations are not always successfully identified due to either the fact that they may reside in uncaptured regions (*i.e*., non-coding regions, regulatory regions or un-annotated coding sequences that are not captured by the capture design) or to biases in standard mapping and variant calling procedures. Therefore, further improvements are required in the development of software tools in order to better deal with regions of the genome that are difficult to map (e.g., paralogous sequences and GC-rich regions). The design of exome capture kits must also be improved to extend the set of captured regions. Alternatively, whole genome sequencing may also be a way to identify mutations in regions not captured by whole exome sequencing.

## 5. Infectious Screens

Establishing an ENU mutagenesis program with the aim of identifying genes involved in the host response to pathogens presents particular challenges. The first is the choice of a pathogen relevant to human health. Mouse models of infection with this pathogen must be available and representative of the corresponding human pathology. Also, the contribution of genetic factors in human and/or mouse response to this pathogen must be proven to support the feasibility of a genetic screen. The second challenge is the choice of the inbred mouse strain to be used for mutagenesis. There is ample evidence that the ENU sensitivity of inbred mice is genetically controlled and thus widely variable across strains [63]. This must be balanced with the varying susceptibility or resistance of inbred strains to infection with specific pathogens. The third challenge is the choice of the screening phenotype. Cell-based phenotypes have been used successfully to identify fundamental mechanisms of innate and acquired immunity [36,37]. The findings, however, require further validation in mouse models to determine a possible role in the infectious process. A clinically relevant, robust, and unequivocal *in vivo* phenotype is also attractive, as it will lead to the identification of the most important molecular determinants for a given infection; it will also minimize the appearance of false positives. Such phenotypes include severe disease (in terms of clinical evaluation or pathogen load) or death, when the mutagenized strain is resistant, or survival, when the mutagenized strain is innately susceptible, following infection. As presented below both screening approaches have led to the identification of key molecules involved in susceptibility or resistance to infectious diseases caused by parasites, bacteria, and viruses.

## 6. Malaria Parasites

Infecting hundreds of thousands of people every year, malaria is a significant cause of morbidity and mortality in developing countries (www.who.org). Having co-existed with humans for centuries, malaria has exerted a significant selective pressure on the human genome [16,86]. Likely the best-known selection has been the retention of deleterious hemoglobinopathies, such as sickle cell anemia, in malaria endemic regions [87,88]. Other variants associated with reduced susceptibility to malaria infections include those affecting erythrocyte proteins [89,90,91,92,93,94], the scavanger receptor CD36 [95,96], and elements of the host immune response, including human leukocyte antigen (HLA) [97] and tomor necrosis factor-alpha (TNF-α) [98], among others [99,100]. Despite these clear examples, the genetic component influencing the human response to malarial parasites is complex, multigenic, and influenced by various environmental factors, including parasite virulence [101,102,103]. 

Cerebral malaria (CM) is the most severe and lethal complication of *Plasmodium falciparum* infection in humans [104,105]. Prevalent in immunologically naïve children, CM is characterized by high fever and a rapid progression to severe cerebral symptoms including impaired consciousness, seizures, and coma [106,107], resulting in death in about 20% of all cases [16,107]. During CM, parasitized erythrocytes (pRBCs) become trapped within the brain microvasculature [103], triggering a strong pro-inflammatory response [104,105] leading to the activation of the vascular endothelium [106], as well as the recruitment of immune cells and activated platelets [108,109,110]. This host-directed immune response results in the disruption of blood-brain barrier integrity [111], suggesting that CM pathogenesis is at least partially caused by over-activation of the inflammatory response [16,106,107]. By gaining a more thorough understanding of this disease, including of the host genetic factors affecting differences in susceptibility, novel and more effective prophylactic and therapeutic interventions can be developed.

Mice infected with *Plasmodium berghei* ANKA (PbA) have been used as a model of CM (experimental cerebral malaria, ECM). Mice susceptible to ECM develop neurological symptoms between days five to eight post-infection, including ataxia, hind limb paralysis, coma, and death [112]. ECM-resistant mice survive the cerebral malaria phase, but subsequently succumb to hyperparasitemia and resultanting anemia within three weeks post-infection [16]. Informative crosses between mouse strains of varying degrees of susceptibility to PbA have revealed at least nine quantitative trait loci (QTL) that modulate the host response to ECM [113,114,115,116,117,118]. However, these methods have failed to identify the causative genes, due in part to the large size of the genomic region and to the high number of positional candidates under the QTL peaks [119]. By introducing random point-mutations and small deletions within a susceptible genetic background, such as B6, B10, or 129S1, ENU-mutagenesis allows for the interrogation and determination of genes that are involved in resistance to ECM.

### 6.1. Screening for Acquired Resistance to Cerebral Malaria

We have successfully utilized ENU-mutagenesis to identify genes responsible for controlling susceptibility to ECM [119]. Male B6 mice (G0) were mutagenized with the administration of three consecutive i.p. injections of ENU. These G0 males were then bred to wild-type B10, 129S1, or B6 females to establish heterozygous G1 offspring. G1 males were out-crossed a second time to wild-type susceptible females to form the G2 generation. One to two G2 females per pedigree were backcrossed to the paternal G1 to produce G3 offspring, fixing mutations to homozygousity in approximately 25% of all animals (Figure 2A). G3 mice were infected with 10^6^ *Plasmodium berghei* ANKA-parasitized RBCs by intravenous injection. The appearance of neurological symptoms and survival time were used as phenotypic markers of ECM disease [119]. Phenodeviant pedigrees were defined as those exhibiting >17% resistant pups in at least three litters or 10 offspring, whichever came first.

Enhanced laboratory resources and technological advances have allowed us to implement three variations of the general protocol outlined above. The first screen out-crossed mutagenized G0 males to the B10 genetic background. G3 animals from this cross were phenotyped for ECM-resistance. To facilitate linkage mapping, G1 males identified as segregating an ECM-resistant phenotype were out-crossed to 129S1 wild-type females. The resulting F1s were intercrossed randomly to generate F2 offspring, which were then phenotyped. Pedigrees identified as resistant were then analyzed for linkage analysis using a genome scan. A total of 6062 G3 mice from 244 G1 males were screened, generating nine phenodeviant pedigrees, with a background survival of approximately 2.8%. From this screen, we have identified an ECM protective mutation in *Jak3* (*Jak3^W81R^*) [119]. A cytosolic tyrosine kinase that interacts with the common γc chain of cytokine receptors (IL-2, -4, -7, -9, -15, -21), JAK3 is required for STAT family members dependent transcriptional development and activation of inflammatory pathways in NK, T, and B cells [120]. *Jak3^W81R^* mutants exhibit reduced numbers of NK cells, CD8^+^ T cells, and B cells, as well as severely reduced production levels of IFNγ by CD4^+^ T cells. We also demonstrated that tasocitinib, a JAK3 inhibitor used clinically to treat rheumatoid arthritis (RA) and Crohn’s disease (CD), can reduce neuroinflammation and increase survival of *Jak3^−/+^* heterozygotes in the ECM model [119]. Genetic variants in JAK and STAT family proteins have been identified as causing certain primary immunodeficiencies and are also associated with chronic inflammatory diseases, such as inflammatory bowel disease (IBD), multiple sclerosis (MS), and systemic lupus erythematosus (SLE) in humans [121,122,123].

With respect to the second screen, we have out-crossed the mutagenized G0 males to the 129S1 genetic background. The 129S1 strain produces larger litters, allowing for the generation of larger numbers of G3 animals. Additionally, out-crossing directly to the 129S1 background eliminated the requirement to complete additional out-crossing of phenodeviant animals. Twenty-eight phenodeviant pedigrees were identified following the screening of 7705 G3 animals in 220 pedigrees, with a background survival of almost 8%. An epistatic interaction between the B6 and 129S1 genetic backgrounds on Chromosomes 4 and 1 was identified in 10 of the 28 phenodeviant pedigrees, potentially masking the effect of ENU-mutagenesis [124]. However, several mutations were identified in this screen, including an abrogated splicing mutation of Exon 6 in the winged-helix transcriptional regulator *Foxn1* gene [125] *Foxn1* mouse mutants are athymic and severely immuno-compromised, while human *FOXN1* mutations cause T-cell immunodeficiency [126]. Heterozygosity for the *Foxn1* mutant allele confers partial protection against ECM, suggesting that FOXN1 transcriptional targets may be relevant to reducing neuroinflammation.

The epistatic interaction between the B6 and 129S1 genetic backgrounds highlights both the limitations and advantages of different variations of the ENU-mutagenesis screen. Both the B6 and 129S1 strains are susceptible to *Plasmodium berghei* ANKA infection, developing neurological symptoms between Days 5 and 10 post-infection. However, in over a third of the phenodeviant pedigrees identified in the mixed background screen, an enrichment of B6 alleles on distal chromosome 4 was associated with resistance to ECM. With such a high percentage of phenodeviant pedigrees mapping to the same locus, we hypothesized that the likelihood of this effect being caused by a single causative ENU-induced mutation was minimal, and that this effect was due to genetic background rearrangements. Additional analysis revealed that ECM resistance on Chromosome 4 (*Berghei* resistance locus 8, named *Berr8*,) was being modulated by a second locus on Chromosome 1 (named *Berr7*). Although we had expected to discover single point mutations due to ENU-mutagenesis, these results highlight the complex nature of cerebral malaria, as well as the difficulty inherent to finding point mutations that are solely responsible for trait modulation.

Due to improvements in technology and the resulting cost reduction, we switched from linkage analysis to exome sequencing analysis for the identification of ENU-induced mutations, removing the requirement for genetic background variations. Hence, the third and final screen was executed on a pure B6 genetic background, wherein the mutagenized G0 males were outcrossed to wild-type B6 females. Switching to the pure genetic background eliminated the likelihood of epistatic interactions between genetic backgrounds, as exhibited in the reduction of background survival rate from almost 8% in the B6x129S1 screen to less than 5% in the pure B6 screen. However, due to smaller litter sizes, almost 40% fewer G3 animals were produced from the 109 screened pedigrees. Even so, eight phenodeviant pedigrees were identified and are currently being investigated.

### 6.2. Screening for Acquired Resistance to Blood-Stage Malaria

ENU-mutagenesis has also been used to identify genes implicated in host resistance to blood-stage malaria. A dominant ENU-mutagenesis screen for erythrocyte production and maturation defects linked to malaria resistance identified two mutations in the *Ank1* gene: an alternative splice acceptor mutation resulting in a frameshift mutation and premature stop codon was identified in *Mpl^−/−^* mice mutagenized on a BALB/c background [127], and a single nonsense mutation was identified in mutagenized SJL/J mice [128]. Both mutations result in early truncation of the ANK1 protein, encoded by *Ank1*. Implicated in hereditary spherocytosis, an inherited form of hemolytic anemia, mouse erythrocytes harboring mutations in *Ank1* exhibit increased resistance to *P. chabaudi*, a model of blood stage malaria, potentially due to parasite maturation impairment [127,128].

### 6.3. Conclusion

ENU-mutagenesis has enabled the identification of individual genes involved in modulating the host response to both cerebral and blood-stage malaria. We have identified mutations in host inflammatory genes involved in T cell development and/or function (*Jak3* and *Foxn1*), thymus development, and immune cell function [119,125]. These results are consistent with the current understanding of the role of T cells in cerebral malaria pathogenesis [129,130,131,132]. Additionally, these genes have been associated with the modulation of other models of acute inflammation as well as of chronic inflammatory conditions [99]. Other labs have identified mutations in the erythrocyte protein ANK1, an important factor in the erythrocyte cytoskeleton [127,128]. Mutations in erythrocytic proteins, including the cell surface Duffy antigen [92] and structural component Band 3 [89,90,91], have been associated with increased resistance to malaria in humans for several years. Together, these findings advance our understanding of the host response to malaria, and may aid in the discovery of novel drug targets against this devastating disease.

## 7. *Salmonella* Bacteria Infections

*Salmonella enterica* infections in humans represent an increasingly significant economic and public health challenge that is associated with high morbidity and mortality in both developing and industrialized countries [133]. In fact, the increase in global population, the emergence of antimicrobial resistance in bacteria, and the prevalence of co-infections (e.g., *Plasmodium*, HIV) exacerbate the burden of this infectious disease [38]. *Salmonella* infection in humans can cause a range of food and waterborne illnesses, from a localized diarrheal disease to the more severe systemic disease, typhoid fever. In fact, nontyphoidal *Salmonella enterica* serovars (e.g., *S*. *typhimurium*, *S*. *enteritidis*) are the second leading cause of bacterial food poisoning in the United States. Importantly, about 1%–4% of these *Salmonella*-infected individuals are at an increased risk of developing sepsis, chronic infection or clinical sequelae (ex. chronic arthritis) [134,135,136]. *Salmonella enterica* Typhi is the etiologic agent of typhoid fever, which is endemic primarily in areas with poor sanitation and a lack of clean drinking water. *Salmonella typhi* causes twenty-one million infections annually, with 220,000 deaths [133]. The outcome of infection depends on the activation of early innate functions, neutrophilic infiltration, phagocytosis by tissue macrophages, and inflammatory cytokine/chemokine secretion (e.g., IFNγ, IL-12, IL-18, TNFα, and IL-6). However, ultimately, the resolution of systemic infection is dependent on both humoral and cell-mediated immune responses [137,138].

In humans, the contribution of host genetics to *Salmonella* infection has been proven by the candidate gene approach and by exome sequencing in patients. Individuals with defects in the IL-12/IL-23 (IL-12β, IL-12Rβ1) and IFNγ (IFNγR1, IFNγR2, STAT1) pathways are in fact predisposed to Mendelian susceptibility to mycobacterial disease (MSMD) and/or disseminated *Salmonella* infection [139,140,141,142,143,144]. Furthermore, major histocompatabilty complex (MHC) class II and III loci, as well as the TNF haplotype, were significantly associated with typhoid fever in a Vietnamese cohort [145]. Although clinical evidence supports a strong role for host genetics, susceptibility to *Salmonella*-related infections is complex and also influenced by environmental factors and bacterial serotype.

*Salmonella typhimurium* infection is a recognized experimental model for studying systemic typhoid-like disease in mice [146,147]. Various classical inbred strains of mice demonstrate differential susceptibility/survival following sub-lethal intravenous infection with *S*. *typhimurium* strain Keller [148]. In particular, the 129 substrains (129S1, 129X1) of mice are highly resistant to virulent infection, compared to DBA/2J mice, which display intermediate mortality, and to the highly susceptible B6 strain. Although the genetic and molecular basis of several mutations important in resistance to *Salmonella* infection in mice have been identified, namely *Nramp1/Slc11a1*, *Tlr4*, and *Pklr*, the low frequency of naturally occurring spontaneous mutations has prompted the use of novel genomic approaches like ENU mutagenesis to identify novel host susceptibility genes to *Salmonella* infection [148,149,150,151,152,153].

### 7.1. Screening for Acquired Susceptibility to Salmonella typhimurium

We used ENU mutagenesis to further decipher the host genetic component of susceptibility to *Salmonella* infection *in vivo*. In the screen, G3 ENU-mutagenized mice between 7 and 9 weeks of age were challenged intravenously through the caudal vein with an infectious dose of virulent *Salmonella typhimurium* strain Keller, varying between 1000 to 10,000 colony forming units (CFUs), depending on the background strains used for breeding. Over the course of 14 days, infected mice were monitored for clinical manifestations of illness including a body score index of less than two, muscle wasting, fur ruffling (fever), inactivity, twirling, and shaking. Susceptible mutants were defined as those presenting severe clinical signs between Days 3 to 7 post-infection (prior to background control mice). On average, a minimum of six to eight G3 mice per G2 female were infected with the expectation of identifying two to five heritable deviant pedigrees following the screening of G3 mice derived from roughly 100 G1 males.

Two prototype breeding schemes differing in the genetic contribution of background strains (B6, 129S1, 129X1, and DBA/2J) have been used in five rounds of screening for *Salmonella* susceptibility. Male 129S1 (G0) mice were mutagenized using a single i.p. injection of 150mg/kg of ENU at 8–10 weeks of age. The first breeding scheme involved the generation of G1 mice produced by two independent G0 males (Figure 2B). The G0 males were crossed to B6 females. For each G1 pedigree, four G2 brother-sister pairs were bred to produce G3 progeny. Using this breeding scheme, the *Salmonella* susceptibility allele *Slc11a1^Asp169^* from B6 mice was segregated into the G2 population. G2 animals carrying the wild-type *Slc11a1* alleles were then selected for further breeding. As the introduction of susceptibility to the B6 background was interfering with our capacity to capture recessive alleles acting in later infection stages (past Day 4), we subsequently modified the breeding scheme as in Figure 2A. Hence in the second round of screening, G0 males were out-crossed to wild-type 129X1 females to generate G1 heterozygote offspring. G1 males were further backcrossed to 129X1 females to generate G2 mice. G2 females were then backcrossed to the G1 male to give rise to G3 progeny, which were then used for primary phenotyping of susceptibility to infection using survival analysis with 10,000 CFUs. Using the following scheme, 643 G3 mice derived from 39 G1 males were screened and two deviant pedigrees were identified: *Oxie & Celie* (*Ity14)* (*Immunity to Typhimurium* locus *14*) and *Jody & Cloe* (*Ity15).* In this particular case, we used a strain that was closely related to the mutagenized males to prevent or minimize the impact of the genetic background on the expressivity of the phenotype while allowing mapping in the G3 animals. We identified 105 SNPs between 129S1 and 129X1. However, their clustering in the genome did not allow the mapping of some pedigrees. Variations of these protocols (Figure 2) were used to facilitate mapping resolution using SNPs between 129S1 and DBA/2J directly in the G3 population. In the third round of screening, G1 males were out-crossed to DBA/2J, and the resulting G2 mice were randomly intercrossed to generate G3 progeny. G3 mice were then screened with an infectious dose of 5000 CFUs. Using this scheme, 1570 G3 mice derived from 65 G1 males were screened, and one deviant pedigree, *Ity16*, was identified, validated, and cloned [154]. In the fourth round of screening, G0 males were out-crossed directly to DBA/2J in order to introduce genetic variability as early as possible in the breeding scheme, thus facilitating mapping (Figure 2B). In this round, 3,348 G3 mice derived from 208 G1 males were screened and four deviant pedigrees were identified: *Cherrie & Walter* (*Ity17*), *Jeanine & Harman (Ity18)*, *Lexie & Leona*, and *Philippe & Desiree*. Lastly, with the onset of whole-exome sequencing as an alternative to mapping using genetic variation between parental strains, the breeding scheme shown in Figure 2B was carried out on an 129S1 background. From the following screen we have infected 580 G3 mice derived from 41 G1 males, and two deviant pedigrees, *Rakeem & Athena* and *Lessie & Virgie*, were identified.

In summary, 8,389 G3 mice derived from 491 G1 males were screened for increased susceptibility to *Salmonella typhimurium* infection as measured by survival analysis. A total of 10 deviant pedigrees have been identified (Table 2). From this screen, we have to date identified, cloned, and characterized *Salmonella* susceptible mutations in *Usp18* (*Usp18^L361F^*), *Ank1* (*Ank1^Gln1357Ter^*), and *Stat4* (*Stat4^G418_E445^*) [154,155,156]. USP18 (Ubiquitin Specific Peptidase 18) both regulates type I IFN signaling and functions as a protease to remove ISG15 adducts from substrate proteins [157,158]. We have reported that decreased survival in mice that carry the *Usp18^L361F^* mutation results from increased bacterial loads in the spleen and liver, as well as increased inflammatory response leading to septic shock [156,159]. In more recent studies, we have shown that regulation of type I IFN signaling is the predominant mechanism affecting the susceptibility of *Usp18^L361F^* mice to bacterial infection. Also, we have found that hyperactivation of type I IFN signaling leads to increased ISGylation and IL-10 production, as well as decreased expression of markers of autophagy [160]. Additionally, we have shown that *Usp18^L361F^* mice are more susceptible to infection with *Mycobacterium tuberculosis* (same as above).

**Table 2:** Summary of the three ENU-mutagenesis screens for experimental cerebral malaria, *Salmonella*, and herpes simplex virus (HSV)-1.

	Malaria	*Salmonella*	HSV-1
G1 males	573	491	265
G3 mice	16,411	8,415	7,802
Deviant pedigrees (in progress)	45	16	11
Confirmed pedigrees	5	3	2

The transcription factor STAT4 (Signal Transducer and Activator of Transcription Factor 4) is a critical mediator of IL-12 signaling. It plays an important role in both innate and adaptive immunity by regulating the transcription of target genes such as *Ifng* and those mediating NK cell cytotoxicity, T helper 1 cell differentiation, and immunoglobulin isotype switching to IgG1. The *Stat4^G418_E445^* mutation results in impaired innate IFNγ secretion, primarily from splenic NK and NKT cells, contributing to increased hepatosplenic bacterial loads. These findings support the importance of the IL-12/IFNγ axis in resistance to *Salmonella* infection.

ANK1 is a structural protein of the erythrocyte membrane, which plays an important role in membrane stability by mediating the attachment of band 3 (SLC4A1) and protein 4.2 (EPB4.2) to the spectrin-based membrane cytoskeleton [161]. Mice homozygous for the *Ank1^Gln1357Ter^* mutation develop hemolytic anemia and present clinicopathological features of human hereditary spherocytosis, the most common form of congenital chronic hemolysis in Europe and North America [162]. On one hand, as observed with other mutations affecting red blood cell turnover [163], *Ank1* deficits protect mice against malaria [128]. On the other hand, normal ANK1 function is critical for an effective host response against infection with *Salmonella*. *Salmonella* susceptibility in *Ank1^Gln1357Ter^* mutant mice is the result of a combination of factors, namely the concomitant deposition of iron in tissues, which favors bacterial growth, and low levels of the iron regulatory hormone hepcidin [154]. In addition, the strong induction of heme oxygenase 1 (*Hmox1*) expression observed during malaria infection and in *Ank1^Gln1357Ter^* mutant results in impaired oxidative burst function, which favors the intracellular replication of bacteria [154,164].

### 7.2. Ex Vivo and in Vivo ENU Screens for Susceptibility to Bacteria Infections

Additional ENU initiatives have uncovered novel genetic determinants of resistance to bacterial infections. Different primary screens in G3 offspring were used, including: (1) measurement of TNF bioactivity after *ex vivo* challenge of thioglycolate-induced peritoneal macrophages with various pathogen-associated molecular patterns (PAMPs) (*Cd36*, *Tnf*, *Map3k8*) [165,166,167]; (2) measurement of type I IFN bioactivity after *ex vivo* challenge of thioglycolate-induced peritoneal macrophages with *Listeria monocytogenes* (*Tmem173/Sting*) [168]; (3) *in vivo* screen for other classes of pathogens (*Slfn2*) [169]; (4) mutations affecting hematopoetic cell development (Genista-*Gfi1*) [170]; and (5) visible phenodeviants presenting inflammatory lesions of the skin (*Scd1*) [171] or of the feet (*Ptpn6/Shp1*) [172]. For example, a TLR2 agonist screen in macrophages identified the *Oblivious* pedigree, which possesses a mutation in *Cd36* resulting in increased susceptibility to infection with Gram positive bacterium *Staphylococcus aureus* [165]. In addition, the *Sluggish* pedigree, which carries a mutation in the *Map3k8* kinase, has impaired type I IFN production downstream of TLR7 and TLR9 signaling, rendering it susceptible to Group B streptococcus infection *in vivo* [166]. Another example is the ENU-induced mutation in *Gfi1* within the *Genista* pedigree, wherein depletion of PMNs confers resistance to *Brucella abortus* infection [173,174] and increased susceptibility to oral infection with *Salmonella typhimurium sfiA*^−^ [170]. Moreover, the *ex vivo* ENU screen using *Listeria monocytogenes* identified the *Goldenticket* pedigree as carrying a mutation in *Tmem173/Sting,* further demonstrating the importance of type I IFN signaling during bacterial infection [168].

### 7.3. Conclusion

ENU-mutagenesis identified single gene effects (novel allele and novel function) within critical pathways involved in immunity to bacterial infection that could potentially be translatable to infection with other classes of pathogens and/or to chronic inflammatory diseases. The findings have emphasized the importance of IFN signaling (*Usp18*, *Stat4*, *Sting*, *Map3k8*) during bacterial infections [155,156,166,168], as well as erythropoeisis and iron metabolism, (*Ank1*) in the case of *Salmonella* pathogenesis [159].

## 8. Herpes Viruses

The *Herpesviridae* family is a large ancient family with a long history of coevolution with their hosts probably predating the origin of the primate lineage. Altogether the nine human herpesviruses infect 90% of the world population causing different types of pathologies that vary considerably according to the immune status of the infected individual. These ubiquitous viruses constitute a striking example of the intricate interplay that can be gradually established between host and pathogen, and show that important information can be gleaned from the study of host-pathogen interactions, namely the contribution of both viral immune evasion and host resistance genes to the outcome of infection.

### 8.1. Cytomegaloviruses

Human cytomegaly virus (HCMV) is the most frequent congenital viral infection in developing countries, potentially leading to blindness, deafness or mental retardation in affected infants. Primary infection or reactivation of the virus can result in severe morbidity and mortality, especially in immune-compromised individuals such as transplant recipients, leukemia or lymphoma patients and AIDS patients. Fortunately, HCMV is closely related to its murine homologue, mouse cytomegalovirus and both cause death in immunocompromised individuals [175,176]. Thus, infection of mice with MCMV represents an excellent model for the study of HCMV pathology and indeed it is an important tool for virologists, immunologists, and geneticists, all of whom have benefited from the well-developed state of the model. Forward genetic studies in inbred mouse strains identified major epistatic (*Klra16*/*H2^k^*) or single gene effects (*Klra7*, *Klra8*) demonstrating the crucial role that natural killer (NK) cell specific activating (Ly49H, Ly49P) and inhibitory (Ly49G) receptors play in response to virus infections (reviewed in [177]). 

### 8.2. Screening for Altered Immune Responses to MCMV

Beutler and colleagues were the ones to initiate the ENU screen for MCMV susceptibility (for the latest review, see [178]). With this strategy, over 20,000 G3 B6 mice carrying ENU mutations were infected i.p. with 10^5^ plaque forming units (pfu) of MCMV. This viral dose was chosen because wild-type B6 mice are uniformly resistant in this infectious experimental situation. However, the pheno-deviant offspring that exhibited clinical signs of disease or/and high viral titers in the spleen were considered susceptible. Several mice with immunodeficiency phenotypes identified from other screens made by Beulter’s group, such as defects in toll-like receptor (TLR) signaling or adaptive immunity, were also tested for their potential MCMV susceptibility. Here, we highlight some of the most important findings that have been made using ENU-mutagenesis to test susceptibility to MCMV infection.

Dendritic cells (DCs) are specialized cells of the hematopoietic system that alert the immune system to the presence of infection. Therefore, they generally represent the first line of defense against pathogens. In the context of MCMV infection, DCs recognize the virus through TLR3 and TLR9, which are able to respectively detect double-stranded RNA (an intermediate product of viral replication) and viral double stranded DNA. Following MCMV recognition, DCs and plasmacytoid DCs (pDCs) in particular, produce large amounts of antiviral type I IFN cytokines (IFN-α/β), which are essential mediators of the innate and adaptive immune responses. Thus, loss-of-function mutations in genes that encode components necessary for the expression of IFN-α/β (such as *Tlr9*, *Tlr3*, *Myd88*, *Trif*, and *Unc93b1*), or that are involved in the IFN-α/β signaling pathway (ie, downstream of IFN-α/β receptor), like *Stat1*, have been shown to increase susceptibility to MCMV infection [179,180,181,182]. It should be noted that among all of these ENU mutations, only the one in *Stat1* was initially identified from the MCMV screen, the others being deduced from immune screens. The NF-κB signaling pathway is also essential for survival to MCMV infection. This is attested by the identification of a loss-of-function mutation in the *Ikbkg* gene encoding NEMO, a regulatory subunit of the IKK complex responsible for the nuclear translocation of NF-κB [183]. *Ex vivo* screens for increased susceptibility to MCMV infection have been performed on peritoneal macrophages isolated from ENU-mutagenized mice, and revealed a missense mutation in the *Eif2ak4* gene encoding GCN2 [184]. This protein is related to PKR, an effector known to inhibit viral replication via phosphorylation of the alpha subunit of eukaryotic initiation factor 2 (eIF2α). The loss-of-function mutation identified in *Eif2ak4* affects the phosphorylation of eIF2α in response to MCMV infection and was therefore associated with an increased susceptibility to MCMV. The MCMV screen, together with the immune screens, led to the identification of several phenodeviants with mutations in genes that contribute to the establishment of an efficient immune response against pathogens, as they act at different levels of IFN-α/β production (TLR9, TRIF and UNC93B1), of IFN-α/β signaling (STAT1), and of the antiviral response (GCN2).

DCs are not the only sites of MCMV recognition. Natural killer (NK) cells are also important responders to MCMV infection, playing a crucial role in containing it at early times post-infection [185,186]. This was initially demonstrated by *in vivo* depletion studies, in which specific antibodies were used to transiently eliminate NK cells before infection with the virus [186,187,188]. Then, the differential susceptibility of the BALB/c and B6 strains was shown to be due to the presence of the NK-activating receptor Ly49H in the latter [189,190]. This receptor engages the MCMV viral protein m157 [191,192], leading to NK cell proliferation and target cell killing [193]. ENU studies allowed the initial discovery of mutations in the *Gimap5* and *Unc13d* genes, in the context of two screens that had been designed to detect *in vivo* defective NK cells and cytotoxic T lymphocyte (CTL) responses [194] and MCMV susceptibility [195], respectively. In both cases, *Gimap5^G38C^* and *Unc13d^jinx/jinx^* were shown to be associated with defects in NK cell activity and impaired resistance to MCMV infection, which are consistent with the crucial function of NK cells in the early control of MCMV replication. *Gimap5^G38C^* affects NK cell development, whereas *Unc13d^jinx/jinx^* NK cells fail to degranulate, a deficit also observed in activated CD8^+^ T cells. Individuals carrying another deleterious mutation, this time in the *Itgb2* gene encoding the integrin β2 CD18, which partially affects NK cell development, are, however, fully resistant to MCMV [196]. In this case, it suggests that even if the β2 integrins are required for optimal NK cell maturation, their partial deficiency could be overcome during MCMV infection, highlighting the robustness of antiviral protective responses.

Other ENU mutations revealed from the screen for host survival against MCMV infection were independently identified in the *Flt3* [197] and *Slfn2* [169] genes. *Flt3^wmfl/wmfl^* mice have been shown to have impaired DC development, making these cells incapable of supporting the effector function of NK cells [197]. In contrast to *Flt3^wmfl/wmfl^*, neither DCs, nor NK cells are impaired in *Slfn2^I135N^* mice [169]. However, both bacterial and viral infections trigger death by apoptosis of peripheral T cells and inflammatory monocytes in *Slfn2^I135N^* mice, indicating the crucial role of Slfn2 in maintaining quiescence in some immune cells. In addition to these ENU mutants recovered from the MCMV screen, four unrelated mutants, called *Mayday*, *Solitaire*, *Goodnight*, and *Slumber*, were shown to die very early post-infection (*i.e*., D2-D3 p.i.) before high viral titers could be observed in the spleen and the liver [198]. Their abrupt death was probably not caused by the direct lytic effects of the virus, but mostly by collateral damage, such as the accompanying inflammatory reaction in response to MCMV infection, since this phenotype was also observed after lipopolysaccharides (LPS) or CpG administration. Based on the comparative sequence analysis of these four mutants, their MCMV susceptibility has been shown to be due to a genetic rearrangement of the *Kcnj8* locus that is likely to have occurred in B6 mice prior to ENU treatment. *Kcnj8* encodes the potassium channel Kir6.1, which maintains the host homeostatic state during the innate immune response. Altogether, these mutations highlight genes that are directly involved in the immune system, but also show the importance of other non-immune signaling pathways, such as homoestasis, in host survival.

### 8.3. Herpes Simplex Virus 1

HSV-1 is the causative agent of herpes simplex encephalitis (HSE), a lethal neurological disease. It is acknowledged that environmental factors have no effect on the pathogenesis of HSE, and no geographical or seasonal patterns in the distribution of the disease have been observed [199,200]. Despite the high seroprevalence of HSV-1 (up to 90%) [201], HSE pathology is rare and affects only a small proportion of otherwise healthy individuals. Therefore, in addition to HSV-1 infection, the second major cause of the disease is the presence of rare host genetic factors, which play a large part in determining the susceptibility of an individual to HSE. Loss-of-function mutations in the *UNC93B1*, *TLR3*, *TRIF*, *TRAF3*, and *TBK1* genes have been associated with a human genetic predisposition to HSE [202,203,204,205,206,207], illustrating the critical role of the UNC93B-TLR3-type I IFN pathway in protection against HSV-1. However, these mutations exhibit incomplete penetrance and represent only a minority of HSE cases. This indicates the likely existence of other anti-HSE pathways and may reflect the effects of additional host genetics factors.

### 8.4. Screening for Acquired Susceptibility to HSE

Two breeding schemes have been used in the mutagenesis screen to identify host susceptibility genes to HSV-1 infection. We started with the B6/B10 screen, where mutagenized B6 G0 males were out-crossed to B10. This allowed linkage mapping with the use of a panel of 255 B6/B10 polymorphic markers (SNPs) distributed across the genome [208]. We then switched to a pure B6 genetic background to eliminate the likelihood of epistatic interactions between the B6 and B10 genetic backgrounds. In total (Table 2), 7,802 G3 B6 mice carrying ENU mutations derived from 265 G1 males were infected i.p. with 10^4^ pfu of HSV-1 strain 17. This dose led to lethal encephalitis in susceptible A/J mice, whereas wild-type B6 mice remained unaffected. Following infection, the ENU-mutagenized mice were monitored for two weeks. The phenodeviant offsprings that exhibited clinical signs of disease or succumbed to the infection were considered susceptible. Using this strategy, we revealed eleven deviant pedigrees. One of these led to the identification of a premature stop codon (L3X) in the Ptprc gene, which encodes the leukocyte common antigen CD45. Ptprc^L3X^ mutant mice showed reduced numbers of CD3^+^ T and mature follicular B cells, suggesting defects in T and B cell development [209]. In this report, we also demonstrated that CD4^+^ Th1 cells, by producing IFNγ, help CD8^+^ T cell recruitment to prevent the dissemination of HSV-1 into the central nervous system, thus protecting mice from lethal HSV-1 infection. Altogether, our data point to CD45 as the first host component involved in the adaptive immune response that directly contributes to susceptibility to HSV-1 and HSE pathology. We are currently investigating the 10 other deviant pedigrees, which have, once again, shown the crucial role of T cells in host survival, but have also revealed that anti-inflammatory factors are critical to protection against HSV-1-induced encephalitis [210].

## 9. Conclusions and Perspectives

ENU-mutagenesis constitutes an inherently unbiased and powerful approach to the production of new alleles. Technological improvements in high-throughput DNA sequencing, combined with the completion of the mouse genome project [68], have greatly facilitated their identification. The recent introduction of NGS has led to a faster and more efficient identification of ENU mutations, which is particularly helpful for analyzing large mutant collections, especially when mapping data are not available to guide an analysis. New variants generated by ENU-mutagenesis mirror those existing in the human population and also represent a natural complement to null alleles being produced by gene targeting. Finding new ENU-induced alleles will also benefit from the new CRISPR/Cas9 technology. ENU variants, although easier to pinpoint by sequencing, need to be validated experimentally as in any forward genetic approach of gene identification. The CRISPR/Cas9 system appears to be an excellent complement to ENU mutagenesis, allowing candidate point mutations identified by NGS to be efficiently confirmed as causative mutations. The ENU mutagenesis approach has proven to be extremely useful in dissecting the genetic architecture of host defenses against infectious diseases. The approach promises to remain current in the field, being constantly renewed by technological advances such as NGS or genome editing.

As summarized in Table 2, over 30,000 G3 mice were screened by our group for either resistance to *Plasmodium berghei* or susceptibility to *Salmonella typhimurium* and HSV-1 infection. In total, 72 deviant pedigrees have been identified and we have to date confirmed ENU-induced mutations for 10 pedigrees. These mutations highlight gene functions that are directly involved in the immune system (*Foxn1*, *Jak3*, *Stat4*, *Usp18* and *Ptprc*), but also show the importance of other non-immune pathways, such as erythropoeisis and iron metabolism (*Ank1*), in host survival (Table 3). Beutler and colleagues also used the ENU mutagenesis approach, and over 20,000 G3 mice were screened for their susceptibility to MCMV. In parallel, they also developed several “immune” ENU screens, where some phenodeviant pedigrees, characterized by defects in the TLR signaling pathway and/or in T/NK cells functions, were then tested for their potential MCMV susceptibility. Of these, it should be noted that among the ENU mutations identified by the group of Beutler, only few were initially revealed by the MCMV *in vivo* screen (*Stat1*, *Unc13d*, *Flt3* and *Slfn2*), the others being deduced from other screens (*Tlr9*, *Trif*, *Unc93b1*, *Ikbkg*, *Eif2ak4*, *Gimap5*) [178]. This observation can be explained by the fact that *in vivo* models are more complex than *in vitro* systems. Indeed, deficiencies in one particular immune cell or signaling pathway can be compensated by the presence of other competent immune cells, making the identification of defective alleles more difficult *in vivo*.

The ENU mutations identified in *Jak3* (*Jak3^W81R^*) and *Ptprc* (*Ptprc^L3X^)* highlighted the critical nature of T cell function for CM pathogenesis and protection against HSV1 infection, respectively. The robustness of these mouse models of neuroinflammation and their ability to detect genetic effects regulating common pathways critical for neuroinflammation are highlighted by the complementary observations that the *Jak3^W81R^* mutant allele (protective in the ECM screen) confers susceptibility to HSV encephalitis (HSE), while the *Ptprc^L3X^* (causing susceptibility to HSE screen) is protective in the ECM model [211]. This approach could be generalized to other interesting pedigrees, where the role of the ENU mutations could be assessed in these different mouse models of infectious diseases. By cross-testing these mutant pedigrees, it should be possible to reveal common and specific pathways, as well as cells and proteins, that are crucial in the protection against malaria and *Salmonella* or viral infections. Moreover, the role of ENU mutations identified in the neuroinflammatory models of ECM and HSE could also be tested in other models of inflammation, such as the model of experimental encephalitis (EAE) that mimics MS, or DSS colitis that models IBD. Preliminary experiments using the EAE model have already suggested that *Ptprc^L3X^* mice are more resistant to EAE symptoms than wild-type and heterozygous littermate controls [210]. Thus, the cross-testing of these mutant pedigrees in different models of inflammation may provide additional information on the gene function, including its role in the pro- and anti-inflammatory balance. It can also provide novel targets for the development of new drugs that could be used in therapy for acute and chronic inflammatory diseases. As an example, a JAK3 inhibitor, currently in clinical use for the treatment of RA and CD (tasocitinib; Pfizer, New York, NY, USA), has been shown to reduce neuroinflammation and increase survival of *Jak3^−/+^* heterozygotes in our ECM model [119]. Therefore, pharmacological modulation of JAK3 mimics the effect of its genetic inactivation, indicating that the ECM screen can identify novel pharmacological targets for drug discovery.

**Table 3:** Genes and pathways identified in ENU screens described in this review.

Pathway	Gene	Screen	Phenotype	Reference
TLR signaling	*Cd36*	Immunity→*S. aureus*	Susceptible	[165]
	*Map3k8*	Immunity→Group B streptococcus	Susceptible	[166]
	*Ptpn6*	Autoimmunity→*L. monocytogenes*	Susceptible	[172]
	*Tlr9*	Immunity→MCMV	Susceptible	[179]
	*Trif*	Immunity→MCMV	Susceptible	[180]
	*Unc93b1*	Immunity→MCMV	Susceptible	[181]
	*Ikbkg*	Immunity→MCMV	Susceptible	[183]
Type I IFN signal	*Usp18*	*S* . Typhimurium	Susceptible	[156,159]
	*Stat1*	MCMV	Susceptible	[182]
Effector	*Eif2ak4*	MCMV	Susceptible	[184]
Cellular immunity	*Jak3*	*P. Berghei*	Resistant	[119]
	*Foxn1*	*P. Berghei*	Resistant	[125]
	*Stat4*	*S* . Typhimurium	Susceptible	[155,156]
	*Tnf*	Immunity→L.*monocytogenes*	Resistant	[167]
	*Gfi1*	Immunity→*S*. Typhimurium	Susceptible	[170]
	*Gimap5*	Immunity→MCMV	Susceptible	[194]
	*Unc13d*	MCMV	Susceptible	[195]
	*Flt3*	MCMV	Susceptible	[197]
	*Slfn2*	MCMV	Susceptible	[169]
	*Ptprc*	HSV-1	Susceptible	[209]
Red cell cytoskeleton	*Ank1*	*S*. Typhimurium**	Susceptible	[154]
	*Ank1*	P. Chabaudi	Resistant	[127,128]
Homeostasis	*Kcnj8*	MCMV	Susceptible	[198]
Lipid metabolism	*Scd1*	Immunity→*S. Pyogenes*	Susceptible	[171]

One objective of the ENU-mutagenesis approach is to translate and validate knowledge obtained in the mouse infectious context to an improved understanding of human immunity and susceptibility to infection. As a starting point, mouse studies are fundamental for exploring host-pathogen interactions, especially when orthologous human genes exist. One striking example came from the discovery of the ENU-induced mutation in the mouse *Unc93b1* gene that causes susceptibility to MCMV [181]. Based on this finding, the group of JL Casanova identified an autosomal recessive UNC93B deficiency in two human patients with HSE [202]. Furthermore, a survey of the literature has shown that human variants identified in our ECM and HSE screens are risk factors for inflammatory diseases. For example, genetic variants in *JAK* and *STAT* family members have been associated with IBD, MS, RA, and SLE [121,122]. *PTPRC* polymorphisms are associated with autoimmune and inflammatory conditions including MS, SLE, and myasthenia gravis [212]. Thus, the ENU-mutagenesis approach should be continued in combination with GWAS studies, thus providing important insights into the pathways, cells, and proteins that directly impact susceptibility to pathogens, as it constitutes an invaluable resource for identifying novel therapeutic treatments.

## References

[1] Brussow H. (2009). Europe, the bull and the Minotaur: The biological legacy of a Neolithic love story. Environ. Microbiol..

[2] McMichael A.J. (2004). Environmental and social influences on emerging infectious diseases: Past, present and future. Philos. Trans. R. Soc. Lond. B. Biol. Sci..

[3] Casanova J.L., Abel L. (2005). Inborn errors of immunity to infection: The rule rather than the exception. J. Exp. Med..

[4] Fauci A.S., Morens D.M. (2012). The perpetual challenge of infectious diseases. N. Engl. J. Med..

[5] Chapman S.J., Hill A.V. (2012). Human genetic susceptibility to infectious disease. Nat. Rev. Genet..

[6] Cobat A., Orlova M., Barrera L.F., Schurr E. (2013). Host genomics and control of tuberculosis infection. Publ. Health Genet..

[7] Plantinga T.S., Johnson M.D., Scott W.K., Joosten L.A., van der Meer J.W., Perfect J.R., Kullberg B.J., Netea M.G. (2012). Human genetic susceptibility to Candida infections. Med. Mycol..

[8] Keynan Y., Malik S., Fowke K.R. (2013). The role of polymorphisms in host immune genes in determining the severity of respiratory illness caused by pandemic H1N1 influenza. Publ. Health Genet..

[9] Min-Oo G., Gros P. (2005). Erythrocyte variants and the nature of their malaria protective effect. Cell Microbiol..

[10] Lederman M.M., Penn-Nicholson A., Cho M., Mosier D. (2006). Biology of CCR5 and its role in HIV infection and treatment. JAMA.

[11] Lindesmith L., Moe C., Marionneau S., Ruvoen N., Jiang X., Lindblad L., Stewart P., LePendu J., Baric R. (2003). Human susceptibility and resistance to Norwalk virus infection. Nat. Med..

[12] Von Bernuth H., Picard C., Puel A., Casanova J.L. (2012). Experimental and natural infections in MyD88- and IRAK-4-deficient mice and humans. Eur. J. Immunol..

[13] International Human Genome Sequencing (2004). Finishing the euchromatic sequence of the human genome. Nature.

[14] The International HapMap Consortium (2005). A haplotype map of the human genome. Nature.

[15] Hohl T.M. (2014). Overview of vertebrate animal models of fungal infection. J. Immunol. Methods.

[16] Longley R., Smith C., Fortin A., Berghout J., McMorran B., Burgio G., Foote S., Gros P. (2011). Host resistance to malaria: Using mouse models to explore the host response. Mamm. Genet..

[17] Sancho-Shimizu V., Zhang S.Y., Abel L., Tardieu M., Rozenberg F., Jouanguy E., Casanova J.L. (2007). Genetic susceptibility to herpes simplex virus 1 encephalitis in mice and humans. Curr. Opin. Allergy Clin. Immunol..

[18] Wick M.J. (2011). Innate immune control of *Salmonella enterica* serovar Typhimurium: Mechanisms contributing to combating systemic *Salmonella* infection. J. Innate Immun..

[19] Waterston R.H., Lindblad-Toh K., Birney E., Rogers J., Abril J.F., Agarwal P., Agarwala R., Ainscough R., Alexandersson M., An P. (2002). Initial sequencing and comparative analysis of the mouse genome. Nature.

[20] Church D.M., Goodstadt L., Hillier L.W., Zody M.C., Goldstein S., She X., Bult C.J., Agarwala R., Cherry J.L., DiCuccio M. (2009). Lineage-specific biology revealed by a finished genome assembly of the mouse. PLoS Biol..

[21] Guenet J.L. (2011). Animal models of human genetic diseases: Do they need to be faithful to be useful?. Mol. Genet. Genet..

[22] Guan C., Ye C., Yang X., Gao J. (2010). A review of current large-scale mouse knockout efforts. Genesis.

[23] Ayadi A., Birling M.C., Bottomley J., Bussell J., Fuchs H., Fray M., Gailus-Durner V., Greenaway S., Houghton R., Karp N. (2012). Mouse large-scale phenotyping initiatives: Overview of the European Mouse Disease Clinic (EUMODIC) and of the Wellcome Trust Sanger Institute Mouse Genetics Project. Mamm. Genome.

[24] Gaj T., Gersbach C.A., Barbas C.F. (2013). ZFN, TALEN, and CRISPR/Cas-based methods for genome engineering. Trends Biotechnol..

[25] Wang H., Yang H., Shivalila C.S., Dawlaty M.M., Cheng A.W., Zhang F., Jaenisch R. (2013). One-step generation of mice carrying mutations in multiple genes by CRISPR/Cas-mediated genome engineering. Cell.

[26] Blake J.A., Bult C.J., Eppig J.T., Kadin J.A., Richardson J.E. (2014). The Mouse Genome Database: Integration of and access to knowledge about the laboratory mouse. Nucl. Acids Res..

[27] Smith C.M., Finger J.H., Hayamizu T.F., McCright I.J., Xu J., Berghout J., Campbell J., Corbani L.E., Forthofer K.L., Frost J.P. (2014). The mouse Gene Expression Database (GXD): 2014 update. Nucl. Acids Res..

[28] Begley D.A., Krupke D.M., Neuhauser S.B., Richardson J.E., Bult C.J., Eppig J.T., Sundberg J.P. (2012). The Mouse Tumor Biology Database (MTB): A central electronic resource for locating and integrating mouse tumor pathology data. Vet. Pathol..

[29] Wiltshire S.A., Leiva-Torres G.A., Vidal S.M. (2011). Quantitative trait locus analysis, pathway analysis, and consomic mapping show genetic variants of Tnni3k, Fpgt, or H28 control susceptibility to viral myocarditis. J. Immunol..

[30] Di Pietrantonio T., Hernandez C., Girard M., Verville A., Orlova M., Belley A., Behr M.A., Loredo-Osti J.C., Schurr E. (2010). Strain-specific differences in the genetic control of two closely related mycobacteria. PLoS Pathog..

[31] Toth L.A., Trammell R.A., Williams R.W. (2014). Mapping complex traits using families of recombinant inbred strains: An overview and example of mapping susceptibility to Candida albicans induced illness phenotypes. Pathog. Dis..

[32] Boivin G.A., Pothlichet J., Skamene E., Brown E.G., Loredo-Osti J.C., Sladek R., Vidal S.M. (2012). Mapping of clinical and expression quantitative trait loci in a sex-dependent effect of host susceptibility to mouse-adapted influenza H3N2/HK/1/68. J. Immunol..

[33] Ferris M.T., Aylor D.L., Bottomly D., Whitmore A.C., Aicher L.D., Bell T.A., Bradel-Tretheway B., Bryan J.T., Buus R.J., Gralinski L.E. (2013). Modeling host genetic regulation of influenza pathogenesis in the collaborative cross. PLoS Pathog..

[34] Guenet J.L., Bonhomme F. (2003). Wild mice: An ever-increasing contribution to a popular mammalian model. Trends Genet..

[35] Chia R., Achilli F., Festing M.F., Fisher E.M. (2005). The origins and uses of mouse outbred stocks. Nat. Genet..

[36] Keane T.M., Goodstadt L., Danecek P., White M.A., Wong K., Yalcin B., Heger A., Agam A., Slater G., Goodson M. (2011). Mouse genomic variation and its effect on phenotypes and gene regulation. Nature.

[37] Simon M.M., Greenaway S., White J.K., Fuchs H., Gailus-Durner V., Wells S., Sorg T., Wong K., Bedu E., Cartwright E.J. (2013). A comparative phenotypic and genomic analysis of C57BL/6J and C57BL/6N mouse strains. Genet. Biol..

[38] Vidal S.M., Malo D., Marquis J.F., Gros P. (2008). Forward genetic dissection of immunity to infection in the mouse. Annu. Rev. Immunol..

[39] Cook M.C., Vinuesa C.G., Goodnow C.C. (2006). ENU-mutagenesis: Insight into immune function and pathology. Curr. Opin. Immunol..

[40] Hoebe K., Beutler B. (2008). Forward genetic analysis of TLR-signaling pathways: An evaluation. Adv. Drug Deliv. Rev..

[41] Hoyne G.F., Goodnow C.C. (2006). The use of genomewide ENU mutagenesis screens to unravel complex mammalian traits: Identifying genes that regulate organ-specific and systemic autoimmunity. Immunol. Rev..

[42] Oliver P.L., Davies K.E. (2012). New insights into behaviour using mouse ENU mutagenesis. Hum. Mol. Genet..

[43] Russell W.L. (1972). Radiation and chemical mutagenesis and repair in mice. Johns Hopkins Med. J. Suppl..

[44] Jaenisch R. (1976). Germ line integration and Mendelian transmission of the exogenous Moloney leukemia virus. Proc. Natl. Acad. Sci. USA.

[45] Russell L.B., Russell W.L. (1992). Frequency and nature of specific-locus mutations induced in female mice by radiations and chemicals: A review. Mutat. Res..

[46] Russell W.L., Kelly E.M., Hunsicker P.R., Bangham J.W., Maddux S.C., Phipps E.L. (1979). Specific-locus test shows ethylnitrosourea to be the most potent mutagen in the mouse. Proc. Natl. Acad. Sci. USA.

[47] Russell W.L., Hunsicker P.R., Carpenter D.A., Cornett C.V., Guinn G.M. (1982). Effect of dose fractionation on the ethylnitrosourea induction of specific-locus mutations in mouse spermatogonia. Proc. Natl. Acad. Sci. USA.

[48] Hitotsumachi S., Carpenter D.A., Russell W.L. (1985). Dose-repetition increases the mutagenic effectiveness of N-ethyl-N-nitrosourea in mouse spermatogonia. Proc. Natl. Acad. Sci. USA.

[49] Singer B. (1976). All oxygens in nucleic acids react with carcinogenic ethylating agents. Nature.

[50] Bignami M., Vitelli A., di Muccio A., Terlizzese M., Calcagnile A., Zapponi G.A., Lohman P.H.M., den Engelse L., Dogliotti1 E. (1988). Relationship between specific alkylated bases and mutations at two gene loci induced by ethylnitrosourea and diethyl sulfate in CHO cells. Mutat. Res..

[51] Van Zeeland A.A. (1988). Molecular dosimetry of alkylating agents: Quantitative comparison of genetic effects on the basis of DNA adduct formation. Mutagenesis.

[52] Vogel E.W., Natarajan A.T. (1995). DNA damage and repair in somatic and germ cells *in vivo*. Mutat. Res..

[53] Fairfield H., Gilbert G.J., Barter M., Corrigan R.R., Curtain M., Ding Y.M., Ascenzo M.D., Gerhardt D.J., He C., Huang W.H. (2011). Mutation discovery in mice by whole exome sequencing. Genet. Biol..

[54] Andrews T.D., Whittle B., Field M.A., Balakishnan B., Zhang Y., Shao Y., Cho V., Kirk M., Singh M., Xia Y. (2012). Massively parallel sequencing of the mouse exome to accurately identify rare, induced mutations: An immediate source for thousands of new mouse models. Open Biol..

[55] Bull K.R., Rimmer A.J., Siggs O.M., Miosge L.A., Roots C.M., Enders A., Bertram E.M., Crockford T.L., Whittle B., Potter P.K. (2013). Unlocking the bottleneck in forward genetics using whole-genome sequencing and identity by descent to isolate causative mutations. PLoS Genet..

[56] Lewis M.A., Quint E., Glazier A.M., Fuchs H., de Angelis M.H., Langford C., van Dongen S., Abreu-Goodger C., Piipari M., Redshaw N. (2009). An ENU-induced mutation of miR-96 associated with progressive hearing loss in mice. Nat. Genet..

[57] Masuya H., Sezutsu H., Sakuraba Y., Sagai T., Hosoya M., Kanedaa H., Miuraa I., Kobayashia K., Sumiyamad K., Shimizu A. (2007). A series of ENU-induced single-base substitutions in a long-range cis-element altering sonic hedgehog expression in the developing mouse limb bud. Genomics.

[58] Arnold C.N., Barnes M.J., Berger M., Blasius A.L., Brandl K., Croker B., Crozat K., Du X., Eidenschenk C., Georgel P. (2012). ENU-induced phenovariance in mice: Inferences from 587 mutations. BMC Res. Notes.

[59] Puk O., Moller G., Geerlof A., Krowiorz K., Ahmad N., Wagner S., Adamski J., de Angelis M.H., Graw J. (2011). The pathologic effect of a novel neomorphic Fgf9(Y162C) allele is restricted to decreased vision and retarded lens growth. PLoS ONE.

[60] Caspary T. (2010). Phenotype-driven mouse ENU mutagenesis screens. Methods Enzymol..

[61] Probst F.J., Justice M.J. (2010). Mouse mutagenesis with the chemical supermutagen ENU. Methods Enzymol..

[62] Georgel P., Du X., Hoebe K., Beutler B. (2008). ENU mutagenesis in mice. Methods Mol. Biol..

[63] Justice M.J., Carpenter D.A., Favor J., Neuhauser-Klaus A., de Angelis M.H., Soewarto D., Moser A., Cordes S., Miller D., Chapman V. (2000). Effects of ENU dosage on mouse strains. Mamm. Genet..

[64] Bainbridge M.N., Wang M., Burgess D.L., Kovar C., Rodesch M.J., D’Ascenzo M., Kitzman J., Wu Y.-Q., Newsham I., Richmond T.A. (2010). Whole exome capture in solution with 3 Gbp of data. Genet. Biol..

[65] Choi M., Scholl U.I., Ji W., Liu T., Tikhonova I.R., Zumbob P., Nayirc A., Bakkaloğlud A., Özend S., Sanjad S. (2009). Genetic diagnosis by whole exome capture and massively parallel DNA sequencing. Proc. Natl. Acad. Sci. USA.

[66] Ng S.B., Buckingham K.J., Lee C., Bigham A.W., Tabor H.K., Dent K.M., Huff C.D., Shannon P.T., Jabs E.W., Nickerson D.A. (2010). Exome sequencing identifies the cause of a mendelian disorder. Nat. Genet..

[67] Pruitt K.D., Harrow J., Harte R.A., Wallin C., Diekhans M., Maglott D.R., Searle S., Farrell C.M., Loveland J.E., Ruef B.J. (2009). The consensus coding sequence (CCDS) project: Identifying a common protein-coding gene set for the human and mouse genomes. Genet. Res..

[68] Mouse Genome Project. http://www.sanger.ac.uk/resources/mouse/genomes/.

[69] DePristo M.A., Banks E., Poplin R., Garimella K.V., Maguire J.R., Hartl C., Philippakis A.A., del Angel G., Rivas M.A., Hanna M. (2011). A framework for variation discovery and genotyping using next-generation DNA sequencing data. Nat. Genet..

[70] Pipeline Space Home. https://biowiki.atlassian.net/wiki/display/PS/Pipeline+Space+Home.

[71] GATK Best Practices. http://www.broadinstitute.org/gatk/guide/best-practices.

[72] Lohse M., Bolger A.M., Nagel A., Fernie A.R., Lunn J.E., Stitt M., Usadel B. (2012). RobiNA: A user-friendly, integrated software solution for RNA-Seq-based transcriptomics. Nucl. Acids Res..

[73] Li H., Durbin R. (2010). Fast and accurate long-read alignment with Burrows—Wheeler transform. Bioinformatics.

[74] Li H., Homer N. (2010). A survey of sequence alignment algorithms for next-generation sequencing. Br. Bioinform..

[75] Li H., Handsaker B., Wysoker A., Fennell T., Ruan J., Homer N., Marth G., Abecasis G., Durbin R. (2009). The Sequence Alignment/Map format and SAMtools. Bioinformatics.

[76] Skliris G.P., Rowan B.G., Al-Dhaheri M., Williams C., Troup S., Begic S., Parisien M., Watson P.H., Murphy L.C. (2009). Immunohistochemical validation of multiple phospho-specific epitopes for estrogen receptor alpha (ERalpha) in tissue microarrays of ERalpha positive human breast carcinomas. Breast Cancer Res. Treat.

[77] Picard. http://picard.sourceforge.net.

[78] Garrison E., Marth G. Haplotype-based variant detection from short-read sequencing. http://arxiv.org/abs/1207.3907.

[79] Nielsen R., Paul J.S., Albrechtsen A., Song Y.S. (2011). Genotype and SNP calling from next-generation sequencing data. Nat. Rev. Genet..

[80] Moresco E.M., Li X., Beutler B. (2013). Going forward with genetics: Recent technological advances and forward genetics in mice. Am. J. Pathol..

[81] Cingolani P., Platts A., Wang le L., Coon M., Nguyen T., Land S.J., Lu X., Ruden D.M. (2012). A program for annotating and predicting the effects of single nucleotide polymorphisms, SnpEff: SNPs in the genome of Drosophila melanogaster strain w1118; iso-2; iso-3. Fly (Austin).

[82] McLaren W., Pritchard B., Rios D., Chen Y., Flicek P., Cunningham F. (2010). Deriving the consequences of genomic variants with the Ensembl API and SNP Effect Predictor. Bioinformatics.

[83] Sherry S.T., Ward M.H., Kholodov M., Baker J., Phan L., Smigielski E.M., Sirotkin K. (2001). DbSNP: The NCBI database of genetic variation. Nucl. Acids Res..

[84] Robinson J.T., Thorvaldsdottir H., Winckler W., Guttman M., Lander E.S., Getz G., Mesirov J.P. (2011). Integrative genomics viewer. Nat. Biotechnol..

[85] Sanger F., Nicklen S., Coulson A.R. (1977). DNA sequencing with chain-terminating inhibitors. Proc. Natl. Acad. Sci. USA.

[86] Kwiatkowski D.P. (2005). How malaria has affected the human genome and what human genetics can teach us about malaria. Am. J. Hum. Genet..

[87] Ayi K., Turrini F., Piga A., Arese P. (2004). Enhanced phagocytosis of ring-parasitized mutant erythrocytes: A common mechanism that may explain protection against falciparum malaria in sickle trait and beta-thalassemia trait. Blood.

[88] Friedman M.J. (1978). Erythrocytic mechanism of sickle cell resistance to malaria. Proc. Natl. Acad. Sci. USA.

[89] Allen S.J., O’Donnell A., Alexander N.D., Mgone C.S., Peto T.E., Clegg J.B., Alpers M.P., Weatherall D.J. (1999). Prevention of cerebral malaria in children in Papua New Guinea by southeast Asian ovalocytosis band 3. Am. J. Trop. Med. Hyg..

[90] Foo L.C., Rekhraj V., Chiang G.L., Mak J.W. (1992). Ovalocytosis protects against severe malaria parasitemia in the Malayan aborigines. Am. J. Trop. Med. Hyg..

[91] Genton B., al-Yaman F., Mgone C.S., Alexander N., Paniu M.M., Alpers M.P., Mokela D. (1995). Ovalocytosis and cerebral malaria. Nature.

[92] Miller L.H., Mason S.J., Clyde D.F., McGinniss M.H. (1976). The resistance factor to Plasmodium vivax in blacks. The Duffy-blood-group genotype, FyFy. N. Engl. J. Med..

[93] Ruwende C., Khoo S.C., Snow R.W., Yates S.N., Kwiatkowski D., Gupta S., Warn P., Allsopp C.E., Gilbert S.C., Peschu N. (1995). Natural selection of hemi- and heterozygotes for G6PD deficiency in Africa by resistance to severe malaria. Nature.

[94] Tishkoff S.A., Varkonyi R., Cahinhinan N., Abbes S., Argyropoulos G., Destro-Bisol G., Drousiotou A., Dangerfield B., Lefranc G., Loiselet J. (2001). Haplotype diversity and linkage disequilibrium at human G6PD: Recent origin of alleles that confer malarial resistance. Science.

[95] Aitman T.J., Cooper L.D., Norsworthy P.J., Wahid F.N., Gray J.K., Curtis B.R., McKeigue P.M., Kwiatkowski D., Greenwood B.M., Snow R.W. (2000). Malaria susceptibility and CD36 mutation. Nature.

[96] Omi K., Ohashi J., Patarapotikul J., Hananantachai H., Naka I., Pottere S., Medanad I.M., Miua J., Ball H.J. (2003). CD36 polymorphism is associated with protection from cerebral malaria. Am. J. Hum. Genet..

[97] Hill A.V., Allsopp C.E., Kwiatkowski D., Anstey N.M., Twumasi P., Rowe P.A., Bennett S., Brewster D., McMichael A.J., Greenwood B.M. (1991). Common west African HLA antigens are associated with protection from severe malaria. Nature.

[98] McGuire W., Hill A.V., Allsopp C.E., Greenwood B.M., Kwiatkowski D. (1994). Variation in the TNF-alpha promoter region associated with susceptibility to cerebral malaria. Nature.

[99] Bongfen S.E., Laroque A., Berghout J., Gros P. (2009). Genetic and genomic analyses of host-pathogen interactions in malaria. Trends Parasitol..

[100] Verra F., Mangano V.D., Modiano D. (2009). Genetics of susceptibility to Plasmodium falciparum: From classical malaria resistance genes towards genome-wide association studies. Parasit. Immunol..

[101] Fortin A., Stevenson M.M., Gros P. (2002). Complex genetic control of susceptibility to malaria in mice. Genes Immun..

[102] Hunt N.H., Golenser J., Chan-Ling T., Parekh S., Rae C., Pottere S., Medanad I.M., Miua J., Ball H.J. (2006). Immunopathogenesis of cerebral malaria. Int. J. Parasitol..

[103] Miller L.H., Baruch D.I., Marsh K., Doumbo O.K. (2002). The pathogenic basis of malaria. Nature.

[104] Newton C.R., Hien T.T., White N. (2000). Cerebral malaria. J. Neurol. Neurosurg. Psychiatry.

[105] Tripathi A.K., Sha W., Shulaev V., Stins M.F., Sullivan D.J. (2009). Plasmodium falciparum-infected erythrocytes induce NF-kappaB regulated inflammatory pathways in human cerebral endothelium. Blood.

[106] Brown H., Hien T.T., Day N., Mai N.T., Chuong L.V., Chau T.T., Loc P.P., Phu N.H., Bethell D., Farrar J. (1999). Evidence of blood-brain barrier dysfunction in human cerebral malaria. Neuropathol. Appl. Neurobiol..

[107] Mishra S.K., Newton C.R. (2009). Diagnosis and management of the neurological complications of falciparum malaria. Nat. Rev. Neurol..

[108] Hafalla J.C., Claser C., Couper K.N., Grau G.E., Renia L., de Souza J.B., Riley E.M. (2012). The CTLA-4 and PD-1/PD-L1 inhibitory pathways independently regulate host resistance to Plasmodium-induced acute immune pathology. PLoS Pathog..

[109] Lacerda-Queiroz N., Rodrigues D.H., Vilela M.C., Rachid M.A., Soriani F.M., Sousa L.P., Campos R.D., Quesniaux V.F., Teixeira M.M., Teixeira A.L. (2012). Platelet-activating factor receptor is essential for the development of experimental cerebral malaria. Am. J. Pathol..

[110] Lou J., Lucas R., Grau G.E. (2001). Pathogenesis of cerebral malaria: Recent experimental data and possible applications for humans. Clin. Microbiol. Rev..

[111] De Souza J.B., Riley E.M. (2002). Cerebral malaria: The contribution of studies in animal models to our understanding of immunopathogenesis. Microbes Infect..

[112] Senaldi G., Vesin C., Chang R., Grau G.E., Piguet P.F. (1994). Role of polymorphonuclear neutrophil leukocytes and their integrin CD11a (LFA-1) in the pathogenesis of severe murine malaria. Infect. Immun..

[113] Bagot S., Idrissa Boubou M., Campinom S., Behrschmidt C., Gorgette O., Guénet J.L., Penha-Gonçalves C., Mazier D., Pied S., Cazenave P.A. (2002). Susceptibility to experimental cerebral malaria induced by Plasmodium berghei ANKA in inbred mouse strains recently derived from wild stock. Infect. Immun..

[114] Berghout J., Min-Oo G., Tam M., Gauthier S., Stevenson M.M., Gros P. (2010). Identification of a novel cerebral malaria susceptibility locus (Berr5) on mouse chromosome 19. Genes Immun..

[115] Bopp S.E., Rodrigo E., Gonzalez-Paez G.E., Frazer M., Barnes S.W., Valim C., Watson J., Walker J.R., Schmedt C., Winzeler E.A. (2013). Identification of the Plasmodium berghei resistance locus 9 linked to survival on chromosome 9. Malar. J..

[116] Campino S., Bagot S., Bergman M.L., Almeida P., Sepulveda N., Pied S., Penha-Gonçalves C., Holmberg D., Cazenave P.-A. (2005). Genetic control of parasite clearance leads to resistance to Plasmodium berghei ANKA infection and confers immunity. Genes Immun..

[117] Ohno T., Nishimura M. (2004). Detection of a new cerebral malaria susceptibility locus, using CBA mice. Immunogenetics.

[118] Bopp S.E., Ramachandran V., Henson K., Luzader A., Lindstrom M., Spooner M., Steffy B.M., Suzuki O., Janse C., Waters A.P. (2010). Genome wide analysis of inbred mouse lines identifies a locus containing Ppar-gamma as contributing to enhanced malaria survival. PLoS ONE.

[119] Bongfen S.E., Rodrigue-Gervais I.G., Berghout J., Torre S., Cingolani P., Wiltshire S.A., Leiva-Torres G.A., Letourneau L., Sladek R., Blanchette M. (2012). An N-ethyl-N-nitrosourea (ENU)-induced dominant negative mutation in the JAK3 kinase protects against cerebral malaria. PLoS ONE.

[120] Nosaka T., van Deursen J.M., Tripp R.A., Thierfelder W.E., Witthuhn B.A., McMickle A.P., Doherty P.C., Grosveld G.C., Ihle J.N. (1995). Defective lymphoid development in mice lacking Jak3. Science.

[121] Jostins L., Ripke S., Weersma R.K., Duerr R.H., McGovern D.P., Hui K.Y., Lee J.C., Schumm L.P., Sharma Y., Anderson C.A. (2012). Host-microbe interactions have shaped the genetic architecture of inflammatory bowel disease. Nature.

[122] International Multiple Sclerosis Genetics Consortium (2011). The genetic association of variants in CD6, TNFRSF1A and IRF8 to multiple sclerosis: A multicenter case-control study. PLoS ONE.

[123] Jakkula E., Leppa V., Sulonen A.M., Varilo T., Kallio S., Kemppinen A., Purcell S., Koivisto K., Tienari P., Sumelahti M. (2010). Genome-wide association study in a high-risk isolate for multiple sclerosis reveals associated variants in STAT3 gene. Am. J. Hum. Genet..

[124] Torre S., van Bruggen R., Kennedy J.M., Berghout J., Bongfen S.E., Langat P., Lathrop M., Vidal S.M., Gros P. (2013). Susceptibility to lethal cerebral malaria is regulated by epistatic interaction between chromosome 4 (Berr6) and chromosome 1 (Berr7) loci in mice. Genes Immun..

[125] Torre S., Gros P. (2014).

[126] Pignata C., Fusco A., Amorosi S. (2009). Human clinical phenotype associated with FOXN1 mutations. Adv. Exp. Med. Biol..

[127] Rank G., Sutton R., Marshall V., Lundie R.J., Caddy J., Romeo T., Fernandez K., Mccormack M.P., Cooke B.M., Foote S.J. (2009). Novel roles for erythroid Ankyrin-1 revealed through an ENU-induced null mouse mutant. Blood.

[128] Greth A., Lampkin S., Mayura-Guru P., Rodda F., Drysdale K., Roberts-Thomson M., McMorran B.J., Foote S.J., Burgio G. (2012). A novel ENU-mutation in ankyrin-1 disrupts malaria parasite maturation in red blood cells of mice. PLoS One.

[129] Belnoue E., Kayibanda M., Vigario A.M., Deschemin J.C., van Rooijen N., Viguier M., Snounou G., Rénia L. (2002). On the pathogenic role of brain-sequestered alphabeta CD8+ T cells in experimental cerebral malaria. J. Immunol..

[130] Finley R.W., Mackey L.J., Lambert P.H., Virulent P. (1982). berghei malaria: Prolonged survival and decreased cerebral pathology in cell-dependent nude mice. J. Immunol..

[131] Renia L., Potter S.M., Mauduit M., Rosa D.S., Kayibanda M., Deschemina J., Snounoub G., Grüner A.C. (2006). Pathogenic T cells in cerebral malaria. Int. J. Parasitol..

[132] Grau G.E., Piguet P.F., Engers H.D., Louis J.A., Vassalli P., Lambert P.H. (1986). L3T4+ T lymphocytes play a major role in the pathogenesis of murine cerebral malaria. J. Immunol..

[133] Crump J.A., Luby S.P., Mintz E.D. (2004). The global burden of typhoid fever. Bull. World Health Organ..

[134] Mastroeni P., Maskell D. (2006). Salmonella Infections: Clinical, Immunological, and Molecular Aspects.

[135] Gordon M.A., Banda H.T., Gondwe M., Gordon S.B., Boeree M.J., Walsh A.L., Corkill J.E., Hart C.A., Gilks C.F., Molyneux M.E. (2002). Non-typhoidal *Salmonella* bacteraemia among HIV-infected Malawian adults: High mortality and frequent recrudescence. AIDS.

[136] Majowicz S.E., Musto J., Scallan E., Angulo F.J., Kirk M., O’Brien S.J., Jones T.F., Fazil A., Hoekstra R.M. (2010). The global burden of nontyphoidal *Salmonella* gastroenteritis. Clin. Infect. Dis..

[137] Mittrucker H.W., Kaufmann S.H. (2000). Immune response to infection with *Salmonella typhimurium* in mice. J. Leukoc. Biol..

[138] Dougan G., John V., Palmer S., Mastroeni P. (2011). Immunity to salmonellosis. Immunol. Rev..

[139] Alcais A., Abel L., Casanova J.L. (2009). Human genetics of infectious diseases: Between proof of principle and paradigm. J. Clin. Invest..

[140] Bustamante J., Zhang S.Y., von Bernuth H., Abel L., Casanova J.L. (2008). From infectious diseases to primary immunodeficiencies. Immunol. Allergy Clin. North Am..

[141] Casanova J.L., Fieschi C., Zhang S.Y., Abel L. (2008). Revisiting human primary immunodeficiencies. J. Intern. Med..

[142] De Beaucoudrey L., Samarina A., Bustamante J., Cobat A., Boisson-Dupuis S., Feinberg J., Al-Muhsen S., Jannière L., Rose Y., Desurenaim M. (2010). Revisiting human IL-12Rbeta1 deficiency: A survey of 141 patients from 30 countries. Med. (Baltim.).

[143] Gordon M. (2008). *Salmonella* infections in immunocompromised adults. J. Infect..

[144] Lammas D.A., Casanova J.L., Kumararatne D.S. (2000). Clinical consequences of defects in the IL-12-dependent interferon-gamma (IFN-gamma) pathway. Clin. Exp. Immunol..

[145] Dunstan S.J., Stephens H.A., Blackwell J.M., Duc C.M., Lanh M.N., Dudbridge F., Phuong C.X., Luxemburger C., Wain J., Ho V.A. (2001). Genes of the class II and class III major histocompatibility complex are associated with typhoid fever in Vietnam. J. Infect. Dis..

[146] House D., Bishop A., Parry C., Dougan G., Wain J. (2001). Typhoid fever: Pathogenesis and disease. Curr. Opin. Infect. Dis..

[147] Santos R.L., Zhang S., Tsolis R.M., Kingsley R.A., Adams L.G., Bäumler A.J. (2001). Animal models of *Salmonella* infections: Enteritis *versus* typhoid fever. Microbes Infect..

[148] Roy M.F., Malo D. (2002). Genetic regulation of host responses to *Salmonella* infection in mice. Genes Immun..

[149] Malo D., Vogan K., Vidal S., Hu J., Cellier M., Schurr E., Fuks A., Bumstead N., Morgan K., Gros P. (1994). Haplotype mapping and sequence analysis of the mouse Nramp gene predict susceptibility to infection with intracellular parasites. Genomics.

[150] Qureshi S.T., Lariviere L., Leveque G., Clermont S., Moore K.J., Gros P., Malo D. (1999). Endotoxin-tolerant mice have mutations in Toll-like receptor 4 (Tlr4). J. Exp. Med..

[151] Poltorak A., He X., Smirnova I., Liu M.Y., van Huffel C., Du X., Birdwell D., Alejos E., Silva M., Galanos C. (1998). Eefective LPS signaling in C3H/HeJ and C57BL/10ScCr mice: Mutations in Tlr4 gene. Science.

[152] Vidal S.M., Malo D., Vogan K., Skamene E., Gros P. (1993). Natural resistance to infection with intracellular parasites: Isolation of a candidate for Bcg. Cell.

[153] Roy M.F., Riendeau N., Bedard C., Helie P., Min-Oo G., Turcotte K., Gros P., Canonne-Hergaux F., Malo D. (2007). Pyruvate kinase deficiency confers susceptibility to *Salmonella typhimurium* infection in mice. J. Exp. Med..

[154] Yuki K.E., Eva M.M., Richer E., Chung D., Paquet M., Cellier M., Canonne-Hergaux F., Vaulont S., Vidal S.M., Malo D. (2013). Suppression of hepcidin expression and iron overload mediate *Salmonella* susceptibility in ankyrin 1 ENU-induced mutant. PLoS One.

[155] Eva M.M., Yuki K.E., Dauphinee S.M., Schwartzentruber J.A., Pyzik M., Paquet M., Lathrop M., Majewski J., Vidal S.M., Malo D. (2014). Altered IFN-gamma-mediated immunity and transcriptional expression patterns in N-Ethyl-N-nitrosourea-induced STAT4 mutants confer susceptibility to acute typhoid-like disease. J. Immunol..

[156] Richer E., Prendergast C., Zhang D.E., Qureshi S.T., Vidal S.M., Malo D. (2010). N-ethyl-N-nitrosourea-induced mutation in ubiquitin-specific peptidase 18 causes hyperactivation of IFN-alphass signaling and suppresses STAT4-induced IFN-gamma production, resulting in increased susceptibility to *Salmonella typhimuriu*. J. Immunol..

[157] Malakhova O., Malakhov M., Hetherington C., Zhang D.E. (2002). Lipopolysaccharide activates the expression of ISG15-specific protease UBP43 via interferon regulatory factor 3. J. Biol. Chem..

[158] Kim K.I., Yan M., Malakhova O., Luo J.K., Shen M.F., Zou W., de la Torre J.C., Zhang D. (2006). Ube1L and protein ISGylation are not essential for alpha/beta interferon signaling. Mol. Cell. Biol..

[159] Richer E., Yuki K.E., Dauphinee S.M., Lariviere L., Paquet M., Malo D. (2011). Impact of Usp18 and IFN signaling in *Salmonella*-induced typhlitis. Genes Immun..

[160] Dauphinee S.M., Richer E., Eva M.M., McIntosh F., Paquet M., Dangoor D., Burkart C., Zhang D.E., Gruenheid S., Gros P. (2014). Contribution of increased ISG15, ISGylation and deregulated type I IFN signaling in Usp18 mutant mice during the course of bacterial infections. Genes Immun..

[161] Perrotta S., Gallagher P.G., Mohandas N. (2008). Hereditary spherocytosis. Lancet.

[162] Eber S.W., Gonzalez J.M., Lux M.L., Scarpa A.L., Tse W.T., Dornwell M., Herbers J., Kugler W., Ozcan R., Pekrun A. (1996). Ankyrin-1 mutations are a major cause of dominant and recessive hereditary spherocytosis. Nat. Genet..

[163] Min-Oo G., Fortin A., Tam M.F., Nantel A., Stevenson M.M., Gros P. (2003). Pyruvate kinase deficiency in mice protects against malaria. Nat. Genet..

[164] Cunnington A.J., de Souza J.B., Walther M., Riley E.M. (2012). Malaria impairs resistance to *Salmonella* through heme- and heme oxygenase-dependent dysfunctional granulocyte mobilization. Nat. Med..

[165] Hoebe K., Georgel P., Rutschmann S., Du X., Mudd S., Crozat K., Sovath S., Shame L., Hartung T., Zähringer U. (2005). CD36 is a sensor of diacylglycerides. Nature.

[166] Xiao N., Eidenschenk C., Krebs P., Brandl K., Blasius A.L., Xia Y., Khovananth K., Smart N.G., Beutler B. (2009). The Tpl2 mutation Sluggish impairs type I IFN production and increases susceptibility to group B streptococcal disease. J. Immunol..

[167] Rutschmann S., Hoebe K., Zalevsky J., Du X., Mann N., Dahiyat B.I., Steed P., Beutler B. (2006). PanR1, a dominant negative missense allele of the gene encoding TNF-alpha (Tnf), does not impair lymphoid development. J. Immunol..

[168] Sauer J.D., Sotelo-Troha K., von Moltke J., Monroe K.M., Rae C.S., Brubaker S.W., Hyodo M., Hayakawa Y., Woodward J.J., Portnoy D.A. (2011). The N-ethyl-N-nitrosourea-induced Goldenticket mouse mutant reveals an essential function of Sting in the *in vivo* interferon response to Listeria monocytogenes and cyclic dinucleotides. Infect. Immun..

[169] Berger M., Krebs P., Crozat K., Li X., Croker B.A., Siggs O.M., Popkin D., Du X., Lawson B.R., Theofilopoulos A.N. (2010). An Slfn2 mutation causes lymphoid and myeloid immunodeficiency due to loss of immune cell quiescence. Nat. Immunol..

[170] Ordonez-Rueda D., Jonsson F., Mancardi D.A., Zhao W., Malzac A., Liang Y., Bertosio E., Grenot P., Blanquet V., Sabrautzki S. (2012). A hypomorphic mutation in the Gfi1 transcriptional repressor results in a novel form of neutropenia. Eur. J. Immunol..

[171] Georgel P., Crozat K., Lauth X., Makrantonaki E., Seltmann H., Sovath S., Hoebe K., Du X., Rutschmann S., Jiang Z. (2005). A toll-like receptor 2-responsive lipid effector pathway protects mammals against skin infections with gram-positive bacteria. Infect. Immun..

[172] Croker B.A., Lawson B.R., Rutschmann S., Berger M., Eidenschenk C., Blasius A.L., Moresco E.M.Y., Sovath S., Cengia L., Shultz L.D. (2008). Inflammation and autoimmunity caused by a SHP1 mutation depend on IL-1, MyD88, and a microbial trigger. Proc. Natl. Acad. Sci. USA.

[173] Jaeger B.N., Donadieu J., Cognet C., Bernat C., Ordonez-Rueda D., Barlogis V., Mahlaoui N., Fenis A., Narni-Mancinelli E., Beaupain B. (2012). Neutrophil depletion impairs natural killer cell maturation, function, and homeostasis. J. Exp. Med..

[174] Barquero-Calvo E., Martirosyan A., Ordonez-Rueda D., Arce-Gorvel V., Alfaro-Alarcon A., Lepidi H., Malissen B., Malissen M., Gorvel J., Moreno E. (2013). Neutrophils exert a suppressive effect on Th1 responses to intracellular pathogen Brucella abortus. PLoS Pathog..

[175] Krmpotic A., Bubic I., Polic B., Lucin P., Jonjic S. (2003). Pathogenesis of murine cytomegalovirus infection. Microbes Infect..

[176] Rawlinson W.D., Farrell H.E., Barrell B.G. (1996). Analysis of the complete DNA sequence of murine cytomegalovirus. J. Virol..

[177] Pyzik M., Gendron-Pontbriand E.M., Vidal S.M. (2011). The impact of Ly49-NK cell-dependent recognition of MCMV infection on innate and adaptive immune responses. J. Biomed. Biotechnol..

[178] Moresco E.M., Beutler B. (2011). Resisting viral infection: The gene by gene approach. Curr. Opin. Virol..

[179] Tabeta K., Georgel P., Janssen E., Du X., Hoebe K., Crozat K., Mudd S., Shamel L., Sovath S., Goode J. (2004). Toll-like receptors 9 and 3 as essential components of innate immune defense against mouse cytomegalovirus infection. Proc. Natl. Acad. Sci. USA.

[180] Hoebe K., Du X., Georgel P., Janssen E., Tabeta K., Kim S.O., Goode J., Lin P., Mann N., Mudd S. (2003). Identification of Lps2 as a key transducer of MyD88-independent TIR signalling. Nature.

[181] Tabeta K., Hoebe K., Janssen E.M., Du X., George P., Crozat K., Mudd S., Mann N., Sovath S., Goode J. (2006). The Unc93b1 mutation 3d disrupts exogenous antigen presentation and signaling via Toll-like receptors 3, 7 and 9. Nat. Immunol..

[182] Crozat K., Georgel P., Rutschmann S., Mann N., Du X., Hoebe K., Beutlerm B. (2006). Analysis of the MCMV resistome by ENU mutagenesis. Mamm. Genome.

[183] Siggs O.M., Berger M., Krebs P., Arnold C.N., Eidenschenk C., Huberb C., Piriea E., Smarta N.G., Khovanantha K., Xia Y. (2010). A mutation of Ikbkg causes immune deficiency without impairing degradation of IkappaB alpha. Proc. Natl. Acad. Sci. USA.

[184] Won S., Eidenschenk C., Arnold C.N., Siggs O.M., Sun L., Brandla K., Mullenb T., Nemerowb G.R., Morescoa E.M.Y., Beutler B. (2012). Increased susceptibility to DNA virus infection in mice with a GCN2 mutation. J. Virol..

[185] Biron C.A. (1999). Initial and innate responses to viral infections—Pattern setting in immunity or disease. Curr. Opin. Microbiol..

[186] Bukowski J.F., Woda B.A., Habu S., Okumura K., Welsh R.M. (1983). Natural killer cell depletion enhances virus synthesis and virus-induced hepatitis *in vivo*. J. Immunol..

[187] Bukowski J.F., Woda B.A., Welsh R.M. (1984). Pathogenesis of murine cytomegalovirus infection in natural killer cell-depleted mice. J. Virol..

[188] Welsh R.M., Dundon P.L., Eynon E.E., Brubaker J.O., Koo G.C., O’Donnell C.L. (1990). Demonstration of the antiviral role of natural killer cells *in vivo* with a natural killer cell-specific monoclonal antibody (NK 1.1). Nat. Immun. Cell Growth Regul..

[189] Brown M.G., Dokun A.O., Heusel J.W., Smith H.R., Beckman D.L., Blattenberger E.A., Dubbelde C.E., Stone L.R., Scalzo A.A., Yokoyama W.M. (2001). Vital involvement of a natural killer cell activation receptor in resistance to viral infection. Science.

[190] Lee S.H., Girard S., Macina D., Busa M., Zafer A., Belouchi A., Gros P., Vidal S.M. (2001). Susceptibility to mouse cytomegalovirus is associated with deletion of an activating natural killer cell receptor of the C-type lectin superfamily. Nat. Genet..

[191] Arase H., Mocarski E.S., Campbell A.E., Hill A.B., Lanier L.L. (2002). Direct recognition of cytomegalovirus by activating and inhibitory NK cell receptors. Science.

[192] Smith H.R., Heusel J.W., Mehta I.K., Kim S., Dorner B.G., Naidenko O.V., Iizuka K., Furukawa H., Beckman D.L., Pingel J.T. (2002). Recognition of a virus-encodedligand by a natural killer cell activation receptor. Proc. Natl. Acad. Sci. USA.

[193] Dokun A.O., Kim S., Smith H.R., Kang H.S., Chu D.T., Yokoyama W.M. (2001). Specific and nonspecific NK cell activation during virus infection. Nat. Immunol..

[194] Barnes M.J., Aksoylar H., Krebs P., Bourdeau T., Arnold C.N., Xia Y., Khovananth K., Engel I., Sovath S., Lampe K. (2010). Loss of T cell and B cell quiescence precedes the onset of microbial flora-dependent wasting disease and intestinal inflammation in Gimap5-deficient mice. J. Immunol..

[195] Crozat K., Hoebe K., Ugolini S., Hong N.A., Janssen E., Rutschmann S., Mudd S., Sovath S., Vivier E., Beutler B. (2007). Jinx, an MCMV susceptibility phenotype caused by disruption of Unc13d: A mouse model of type 3 familial hemophagocytic lymphohistiocytosis. J. Exp. Med..

[196] Crozat K., Eidenschenk C., Jaeger B.N., Krebs P., Guia S., Beutler B., Vivier E., Ugolini S. (2011). Impact of beta2 integrin deficiency on mouse natural killer cell development and function. Blood.

[197] Eidenschenk C., Crozat K., Krebs P., Arens R., Popkin D., Arnolda C.N., Blasiusa A.L., Benedictb C.A., Morescoa E.M.Y., Xiaa Y. (2010). Flt3 permits survival during infection by rendering dendritic cells competent to activate NK cells. Proc. Natl. Acad. Sci. USA.

[198] Croker B., Crozat K., Berger M., Xia Y., Sovath S., Schaffer L., Eleftherianos I., Imler J., Beutler B. (2007). ATP-sensitive potassium channels mediate survival during infection in mammals and insects. Nat. Genet..

[199] Abel L., Plancoulaine S., Jouanguy E., Zhang S.Y., Mahfoufi N., Nicolas N., Sancho-Shimizu V., Alcaïs A., Guo Y., Cardon A. (2010). Age-dependent Mendelian predisposition to herpes simplex virus type 1 encephalitis in childhood. J. Pediatr..

[200] Yao H.W., Ling P., Chen S.H., Tung Y.Y., Chen S.H. (2012). Factors affecting herpes simplex virus reactivation from the explanted mouse brain. Virology.

[201] Kennedy P.G., Chaudhuri A. (2002). Herpes simplex encephalitis. J. Neurol. Neurosurg. Psychiatry.

[202] Casrouge A., Zhang S.Y., Eidenschenk C., Jouanguy E., Puel A., Yang K., Alcais A., Picard C., Mahfoufi N., Nicolas N. (2006). Herpes simplex virus encephalitis in human UNC-93B deficiency. Science.

[203] Zhang S.Y., Jouanguy E., Ugolini S., Smahi A., Elain G., Romero P., Segal D., Sancho-Shimizu V., Lorenzo L., Puel A. (2007). TLR3 deficiency in patients with herpes simplex encephalitis. Science.

[204] Sancho-Shimizu V., Perez de Diego R., Lorenzo L., Halwani R., Alangari A., Israelsson E., Fabrega S., Cardon A., Maluenda J., Tatematsu M. (2011). Herpes simplex encephalitis in children with autosomal recessive and dominant TRIF deficiency. J. Clin. Invest..

[205] Perez de Diego R., Sancho-Shimizu V., Lorenzo L., Puel A., Plancoulaine S., Picard C., Herman M., Cardon A., Durandy A., Bustamante J. (2010). Human TRAF3 adaptor molecule deficiency leads to impaired Toll-like receptor 3 response and susceptibility to herpes simplex encephalitis. Immunity.

[206] Guo Y., Audry M., Ciancanelli M., Alsina L., Azevedo J., Herman M., Anguiano E., Sancho-Shimizu V., Lorenzo L., Pauwels E. (2011). Herpes simplex virus encephalitis in a patient with complete TLR3 deficiency: TLR3 is otherwise redundant in protective immunity. J. Exp. Med..

[207] Herman M., Ciancanelli M., Ou Y.H., Lorenzo L., Klaudel-Dreszler M., Pauwels E., Sancho-Shimizu V., de Diego R.P., Abhyankar A., Israelsson E. (2012). Heterozygous TBK1 mutations impair TLR3 immunity and underlie herpes simplex encephalitis of childhood. J. Exp. Med..

[208] Xia Y., Won S., Du X., Lin P., Ross C., la Vine D., Wiltshire S., Leiva G., Vidal S.M., Whittle B. (2010). Bulk segregation mapping of mutations in closely related strains of mice. Genetics.

[209] Caignard G., Leiva-Torres G.A., Leney-Greene M., Charbonneau B., Dumaine A., Fodil-Cornu N., Pyzik M., Cingolani P., Schwartzentruber J., Dupaul-Chicoine J. (2013). Genome-wide mouse mutagenesis reveals CD45-mediated T cell function as critical in protective immunity to HSV-1. PLoS Pathog..

[210] Caignard G., Vidal S.M. (2014).

[211] Caignard G., Gros P., Vidal S.M. (2014).

[212] Tchilian E.Z., Beverley P.C. (2006). Altered CD45 expression and disease. Trends Immunol..

